# Novel pyrazole-clubbed triazole scaffolds as promising inhibitors for carbon steel corrosion in sulfuric acid and as antibacterial agents: electrochemical and computational evaluation

**DOI:** 10.1186/s13065-025-01704-x

**Published:** 2026-01-31

**Authors:** Kamelia Belal, A. H. El-Askalany, Eslam A. Ghaith, Ahmed Fathi Salem Molouk

**Affiliations:** 1 Department of Chemistry, Faculty of Science, Mansoura University, Mansoura, 35516 Egypt; 2 Faculty of Science, Mansoura University Sustainable Energy Research Lab (MSER), Mansoura University, Mansoura, 35516 Egypt; 3 Faculty of Science, New Mansoura University, New Mansoura, Egypt

**Keywords:** Metal protection, Carbon steel, H_2_SO_4_, Triazole scaffolds, Antibacterial activity, DFT

## Abstract

Herein, two novel pyrazole-clubbed triazole scaffolds were synthesized, characterized, and evaluated as inhibitors for carbon steel dissolution in 0.5 M H_2_SO_4_ *via* open circuit potential (OCP), potentiodynamic polarization (PP), electrochemical impedance spectroscopy (EIS), potential of zero charge (PZC), surface characterizations, test solution analysis, and DFT studies. Maximum inhibition reached 95.3% and 93.5% for TBF and TMP, respectively, at 298 K. Based on PP, TBF and TMP function as mixed-type inhibitors, reducing current density from 324.0 µA cm^− 2^ to 15.30 and 21.20 µA cm^− 2^, whereas EIS showed an increase in charge transfer resistance from 41.90 Ω cm² to 812.6 and 633.2 Ω cm² for TBF and TMP, respectively. Inhibition peaked at 318 K and then decreased slightly at 328 K, indicating that a stable adsorbed layer had developed on the surface. Adsorption analysis revealed good agreement with the Langmuir isotherm. Following one day of immersion, Atomic force microscopy (AFM), Fourier transform infrared spectroscopy (FT-IR), contact angle (CA), and X-ray photoelectron spectroscopy (XPS) confirmed the adsorption of the inhibitor. UV-visible spectroscopy verified chemical interactions among TBF/TMP and steel. Additionally, both compounds exhibited high antibacterial activity. Experimental findings were further supported *via* DFT computations and Monte Carlo (MC) simulations. The novelty of this study lies in the molecular structure of TBF and TMP, where electron-rich triazole and pyrazole rings are synergistically integrated with extended π-conjugation to enhance surface adsorption and inhibition efficiency. Moreover, the inhibitors were prepared using an eco-friendly, ultrasound-assisted method, combining high performance, cost-effectiveness, and environmental sustainability. Their outstanding thermal stability and superior inhibition efficiency in strongly acidic media underscore the innovative molecular architecture and demonstrate their potential as highly effective inhibitors under harsh conditions. Thus, the synthesized compounds effectively control corrosion, supporting future development of efficient, multifunctional, and eco-friendly inhibitors.

**Graphical abstract:**

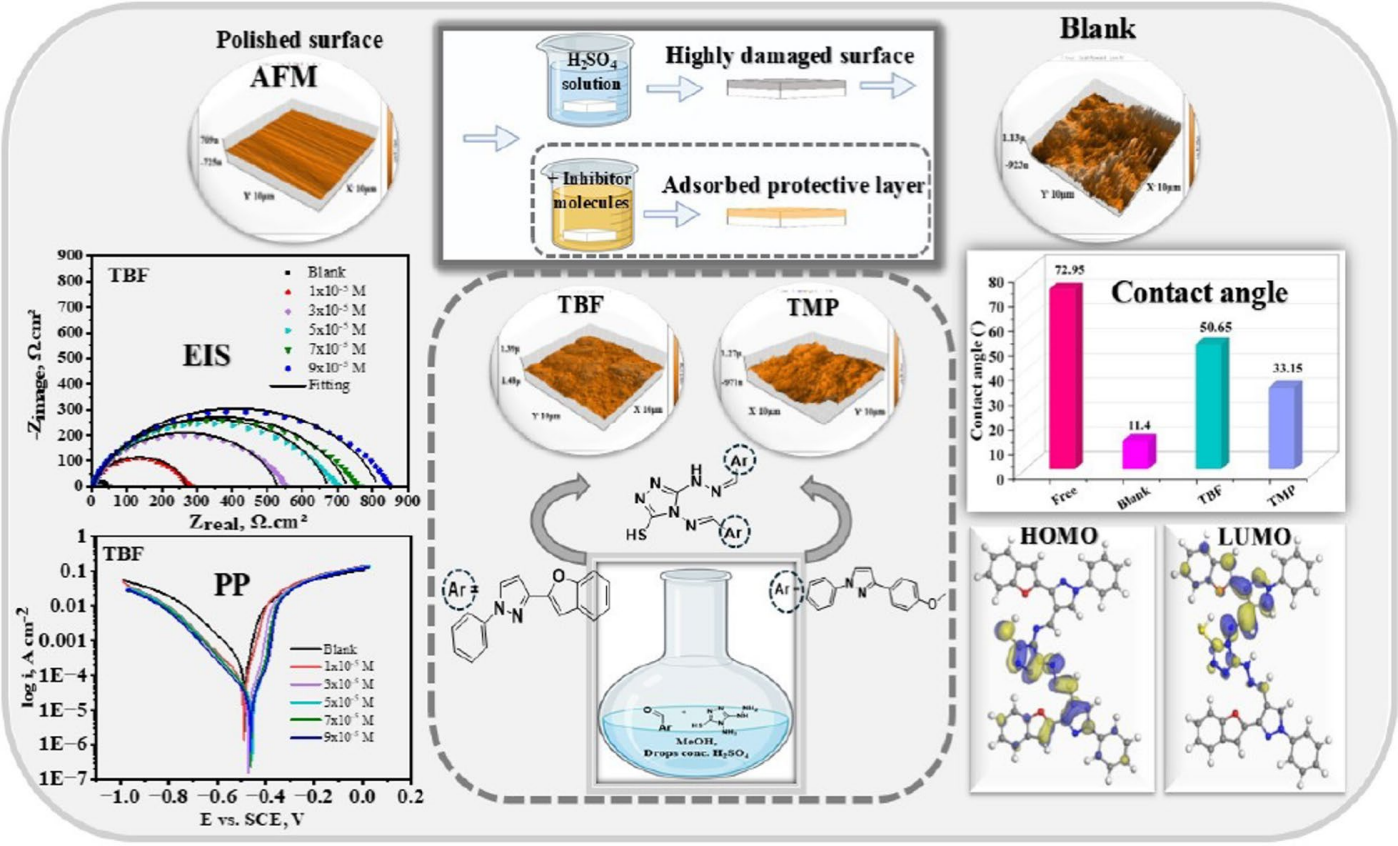

**Supplementary Information:**

The online version contains supplementary material available at 10.1186/s13065-025-01704-x.

## Introduction

 Corrosion is the permanent loss of a metal or alloy’s chemical and physical properties resulting from electrochemically driven deterioration. Globally, this corrosive reaction causes several issues related to human safety and financial loss. Since many significant metals and alloys must be preserved for development under varied conditions and environments, corrosion prevention remains a difficulty and a source of concern [1]. Excellent plasticity, strong weldability, and great mechanical properties are only a few of the remarkable qualities that carbon steel exhibits. It is widely used in various industrial sectors, including water treatment, healthcare, the petroleum and petrochemical industries, and others [2]. But it is extremely prone to corrosion, particularly in acidic situations, which can result in production halts, tank leaks, safety risks, and a shorter lifespan for mechanical equipment [3]. Although it is challenging to halt the dissolution totally, it can be slowed down by employing specific techniques. One such technique is the addition of organic products in slight quantities to acid solutions to impede dissolution. These products are referred to as inhibitors [4]. Employing corrosion inhibitors is an extremely effective way to mitigate corrosion in various materials [5]. Several factors influence the selection of a suitable inhibitor, including its price, capacity to safeguard the metal, and potential environmental impacts. The adsorption concept is how many inhibitors work, producing a protective layer onthemetal. These substances can help reduce metal deterioration during handling, making them very useful in industrial procedures involving acid cleaning [6]. Due to the widespread usage of sulphuric acid in pickling and descaling processes, it was selected as the corrosive medium [7]. Organic compounds with heteroatoms (N, O, and S) have long been recognized as efficient corrosion inhibitors among various types of corrosion inhibitors. This is primarily due to their ability to contribute electron density to the metal. This ability to donate electrons facilitates the formation of coordination bonds or electrostatic interactions, leading to the creation of stable layers that prevent corrosive species from entering [8]. Triazoles are unique compounds that have garnered significant interest in the fields of organic synthesis and corrosion inhibition due to their ability to form organometallic complexes with metal surfaces through the delocalization of electrons on nitrogen atoms and π-systems, which serve as barriers to corrosive solutions [9–11]. These substances have garnered considerable attention as flexible scaffolds that can be used in place of conventional organic molecules. Triazoles are favored for their low toxicity and antibacterial qualities in addition to their superior corrosion inhibition characteristics, which make them appealing options for environmentally friendly and long-lasting corrosion prevention. Moreover, compounds enclosing triazole moieties are well-known in pharmaceutical applications [12, 13]. Consequently, in recent years, research on these novel triazole derivative inhibitors has gained significant attention. Additionally, pyrazole derivatives are heterocyclic compounds with a wide range of applications. They are nontoxic (used as insecticides, fungicides, herbicides, dyes, and pesticides) and are good candidates for slowing down metal corrosion [14–16]. Researchers are still working to enhance the heterocycle’s role in inhibiting metal dissolution. Adding more functional groups or moieties to their skeleton is one tactic for this. Taking the previous considerations into account, two novel compounds were developed and tailored in the current study to enhance the coordination ability of heteroatoms with the metal surface and reduce carbon steel dissolution. Then, the ability of 4-(((*E*)-(3-(benzofuran-2-yl)-1-phenyl-1*H*-pyrazol-4-yl)methylene)amino)-5-(2-((*E*)-(3-(benzofuran-2-yl)-1-phenyl-1*H*-pyrazol-4-yl)methylene)hydrazinyl)-4*H*-1,2,4-triazole-3-thiol (TBF) and 4-(((*E*)-(3-(4-methoxyphenyl)-1-phenyl-1*H*-pyrazol-4-yl)methylene)amino)-5-(2-((*E*)-(3-(4-methoxyphenyl)-1-phenyl-1*H*-pyrazol-4-yl)methylene)hydrazinyl)-4*H*-1,2,4-triazole-3-thiol (TMP) to hinder carbon steel dissolution was investigated using various methodologies, including electrochemical tests (OCP, EIS, PP, and PZC), surface analyses, and UV-visible spectroscopy to evaluate their anticorrosion performance. Additionally, the antibacterial activity of TBF and TMP has been assessed “in vitro” using the bacterial strains (B. subtilis and E. coli). To verify the data gathered from experimental investigations, quantum chemical computations, and MC simulations were also carried out. The innovation of this study lies in the molecular design strategy, which integrates electron-donating centers within conjugated frameworks to achieve stronger adsorption and enhanced corrosion inhibition performance. Furthermore, the ultrasound-assisted, eco-friendly synthesis route offers a sustainable and cost-effective alternative to conventional preparation methods. The use of an integrated multi-technique approach provides comprehensive insights into the inhibition mechanism. In addition, the remarkable thermal stability and excellent performance of the synthesized scaffolds under high-temperature and strongly acidic conditions highlight their potential as robust and reliable corrosion inhibitors for harsh environments. Overall, this work contributes to the advancement of sustainable, multifunctional triazole-based inhibitors that exhibit both strong anticorrosive and antibacterial properties.

## Experimental techniques

### General procedure for inhibitor preparation

Mixtures of 4-amino-5-hydrazineyl-4*H*-1,2,4-triazole-3-thiol (**1**) (0.146 g, 1 mmol) and 3-(benzofuran-2-yl)-1-phenyl-1*H*-pyrazole-4-carbaldehyde (**2**) (0.576 g, 2 mmol) or 3-(4-methoxyphenyl)-1-phenyl-1*H*-pyrazole-4-carbaldehyde (**3**) (0.556 g, 2 mmol) in MeOH (20 mL) containing a catalytic amount of conc. H_2_SO_4_ was irradiated by ultrasound for 3 min or refluxed as a conventional method for 18–23 min (Table 1). The formed precipitates were collected on hot, filtered, and rinsed *via* hot MeOH (3 × 20 mL). Finally, the precipitates of TBF and TMP compounds were oven-dried at 80 °C. The spectra are provided in the Supplementary File S1-S6.

### Comparison between ultrasound and conventional methods

**Scheme 1:**
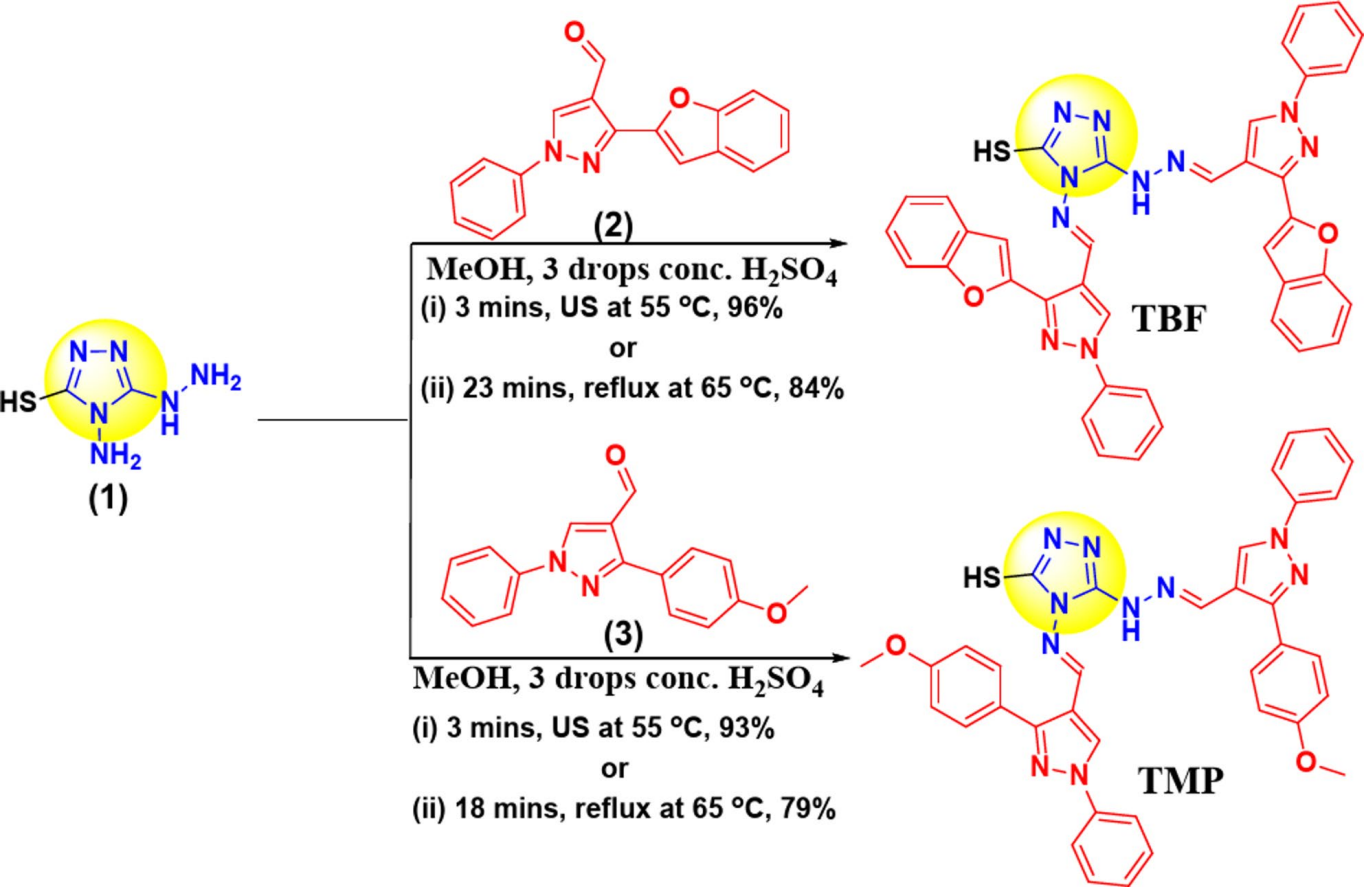
Synthetic approach of the target inhibitors TBF and TMP

The reaction times and quantitative yields of the synthesized hybrids (TBF & TMP) are presented, along with the economic yields and value (YE), which measures the effectiveness of a chemical reaction in terms of both conventional and ultrasound methodologies for the same reaction (Table 1 and Scheme 1). YE calculations were performed through Eq. 1 [13]. Recognizing the significance of ultrasound irradiation as an eco-friendly and alternative methodology, Table 1 confirms the efficiency of the US-assisted synthesis technique, which yields higher productivity (96% and 93%) compared to the conventional technique (84% & 79%) [17].

1 $$\:\mathrm{Y}\mathrm{E}=\frac{\mathrm{Y}\mathrm{i}\mathrm{e}\mathrm{l}\mathrm{d}}{\mathrm{R}\mathrm{e}\mathrm{a}\mathrm{c}\mathrm{t}\mathrm{i}\mathrm{o}\mathrm{n}\:\mathrm{T}\mathrm{i}\mathrm{m}\mathrm{e}}\:\:\:\:\:\:\:$$

**Table 1:** Comparison between conventional and ultrasound irradiation methods for scaffolds TBF and TMP

Compound	Ultrasound Method			Convention Method			Mp (^0^C)
	Time (min)	Yield (%)	YE	Time (min)	Yield (%)	YE	
TBF	3	96	32	23	84	3.6	244–246
TMP	3	93	31	18	79	4.3	247–249

4-(((*E*)-(3-(benzofuran-2-yl)-1-phenyl-1*H*-pyrazol-4-yl)methylene)amino)-5-(2-((*E*)-(3-(benzofuran-2-yl)-1-phenyl-1*H*-pyrazol-4-yl)methylene)hydrazinyl)-4*H*-1,2,4-triazole-3-thiol (TBF). Yellow powder; IR (ν_max_, cm^− 1^, reflectance): 3128 (NH), 1636, 1593, 1544, 1500 (C = N & C = C). ^1^HNMR (DMSO-*d*_6_, 400 M*Hz*): *δ* (ppm) 7.33 (t, *J* = 7.4 *Hz*, 2 H), 7.39–7.44 (m, 3 H), 7.49 (t, *J* = 7.4 *Hz*, 1H), 7.57 (t, *J* = 7.8 *Hz*, 2 H), 7.62 (s, 2 H), 7.65 (s, 1H), 7.67–7.71 (m, 2 H), 7.73–7.77 (m, 2 H), 7.94 (s, 1H), 8.01–8.07 (m, 4 H), 8.82 (s, 1H), 8.99 (s, 1H), 9.45 (s, 1H), 10.78 (s, 1H), 10.81 (s, 1H). 13.53 (s, 1H). ^13^CNMR (DMSO-*d*_*6*_, 100 M*Hz*): *δ* (ppm) 106.28, 107.08, 111.77, 111.82, 116.54, 118. 26, 119.41 (2 C), 119.69 (2 C), 122.03, 122.23, 123.83, 124.09, 125.61, 126.01, 127.76, 128.41 (2 C), 128.64, 128.79, 130.14 (2 C), 130.20, 130.33 (2 C), 138.07, 139.09, 139.32, 141.99, 143.86, 148.71, 148.82, 149.56, 153.72, 154.67, 154.83, 159.90. (EMIS) m/z (%): 686.06 (M^+^, 19.93%), 566.87 (59.73%), 396.10 (56.30%), 367.55 (61.77%), 295.22 (100%, base peak), 285.01 (91.43%), 272.15 (46.50%), 243.18 (69.15%).

4-(((*E*)-(3-(4-methoxyphenyl)-1-phenyl-1*H*-pyrazol-4-yl)methylene)amino)-5-(2-((*E*)-(3-(4-methoxyphenyl)-1-phenyl-1*H*-pyrazol-4-yl)methylene)hydrazinyl)-4*H*-1,2,4-triazole-3-thiol (TMP). Buff powder; IR (ν_max_, cm^− 1^, reflectance): 3336 (NH), 3071,3003 (sp^2^ CH), 2936, 2903 (sp^3^ CH), 1637, 1608, 1577, 1540, 1501 (C = N & C = C). ^1^HNMR (DMSO-*d*_6_, 500 M*Hz*): *δ* (ppm) 3.81 (2CH_3_, 6 H), 7.05–7.08 (m, 4 H), 7.34 (t, *J* = 7.5 *Hz*, 1H), 7.41 (t, *J* = 7.7 *Hz*, 1H), 7.50 (t, *J* = 8 *Hz*, 2H), 7.57 (t, *J* = 8.2 *Hz*, 2H), 7.66 (d, *J* = 8 *Hz*, 2H), 7.77 (d, *J* = 8.5 *Hz*, 2H), 7.96–7.98 (m, 4 H), 8.41 (s, 1H), 8.82 (s, 1H), 9.25 (s, 1H), 10.05 (s, 1H), 10.47 (s, 1H), 13.30 (s, 1H). ^13^CNMR (DMSO-*d*_*6*_, 125 M*Hz*): *δ* (ppm) 55.24, 55.27, 114.16 (2 C), 114.34 (2 C), 114.85, 116.81, 118.63 (2 C), 119.02 (2 C), 123.66, 124.49, 126.45, 126.75, 127.47, 129.37, 129.56 (2 C), 129.60 (2 C), 129.67 (2 C), 129.77 (2 C), 138.14, 138.81, 139.09, 147.99, 151.15, 152.78, 155.77, 159.17, 159.53, 159.93. (EMIS) m/z (%): 666.38 (M^+^, 45.74%), 625.07 (100%, base peak), 621.59 (93.53%), 291.59 (91.87%), 290.79 (88.37%), 256.45 (79.98%), 203.77 (80.15%), 173.12 (95.95%).

### Working electrode and solution preparation

The carbon steel sheet had chemical compositions (wt%) of 0.2% C, 0.5% Mn, 0.25% Si, 0.05% S, and the remaining Fe. It was cut into pieces 1 cm by 1 cm and solidly bonded with epoxy and hardener. This ensured a 1 cm² exposed area for the coupon utilized in the electrochemical tests. Before every test, the working electrode was cleaned in accordance with the established protocol[11]. Diluted sulfuric acid (0.5 M) was employed as a corrosive medium. A stock solution with a 10:90 ratio of dimethyl glyoxime: absolute ethanol was prepared to guarantee the solubility of TBF and TMP. From the stock solution (10^− 3^ M), various concentrations of the tested inhibitors (1–9 × 10^− 5^ M) were prepared in 0.5 M H_2_SO_4_. To eliminate any possible influence of the solvent, the percentage of DMSO and ethanol was kept constant in all test solutions, including the blank tests and those containing inhibitors. Therefore, the observed inhibition effect arises from the tested compounds, not from the solvents.

### Electrochemical measurements

All tests were performed using GAMRY (5000E, USA) potentiostat/galvanostat/ZRA *via* a glass cell with three electrodes: a carbon steel working electrode, a saturated calomel electrode (SCE) as the reference electrode, and a platinum wire serving as the counter electrode. Before each test, the electrodes were left in the tested solutions for 35 min to ensure a stable OCP. For PP studies, the electrode potential was swept from − 500 mV to + 500 mV versus OCP at a scan rate of 0.5 mV/s to generate current-potential graphs. The EIS technique was also carried out at the E_OCP_ using a 10-mV signal amplitude over a frequency range of 10^5^ *Hz* to 0.1 *Hz*.

PZC was evaluated via EIS over a range of applied potentials with an AC perturbation of 10 mV. The dependence of C_dl_ on the applied potential was plotted, and the PZC was identified at the potential corresponding to the minimum C_dl_ value. Three replications of each experiment were conducted to guarantee accuracy.

### Surface studies

A morphological analysis of carbon steel samples was conducted using Nanosurf FlexAFM 3. The surface was pretreated in the same manner as the working electrode and immersed for 24 h in 0.5 M H_2_SO_4_, both with and without the optimum concentrations of TBF and TMP at 298 K. Samples were collected and dried before being analyzed using AFM. The chemical interactions of the inhibitors’ functional group over carbon steel were characterized *via* FT-IR analysis. A water contact angle investigation was conducted to determine the specimen’s surface hydrophobicity. Further, for XPS analysis, the elements observed on the surface were analyzed using AXIX Ultra DLD, Kratos, UK.

### UV–Visible spectra

UV/Visible spectra of the tested inhibitors and the test solutions incorporating the inhibitor after immersion of carbon steel for 2 days were recorded using a UV/vis spectrometer (T80, UK).

### Antibacterial activity test

The antibacterial activity of TBF and TMP was assessed *via* the agar well diffusion method [18]. The microbial inoculum is spread evenly over the surface of the agar plate to prepare it. After that, a sterile cork borer or pipette tip is used under aseptic conditions to create a 9 mm well. Then, 100 µL of the sample at the necessary concentration is added to it. After that, the plates are incubated in an environment suitable for the microbe under investigation. Following incubation, the diameter of the inhibitory zones of the antibacterial agents was measured.

### Computational studies

DFT calculations were performed *via* the Dmol³ module in BIOVIA Materials Studio 2017 to optimize the geometries of TBF and TMP. The molecular and electronic structures of the compounds govern their effectiveness and corrosion-inhibition behavior [19]. The GGA, BOP functional, and DNP basis set were applied in an aqueous environment [20]. According to the literature [21, 22], several quantum parameters were computed.

The interactions that occur between molecular entities and the carbon steel substrate were clarified using the MC simulation [23].The adsorption configurations and interaction energies were determined using the Adsorption Locator module. The Fe (110) surface is chosen due to its packed area and high stabilizing energy [24]. The COMPASS force field optimized every structure that made up the system [25].

## Results and discussion

### OCP

**Fig. 1:**
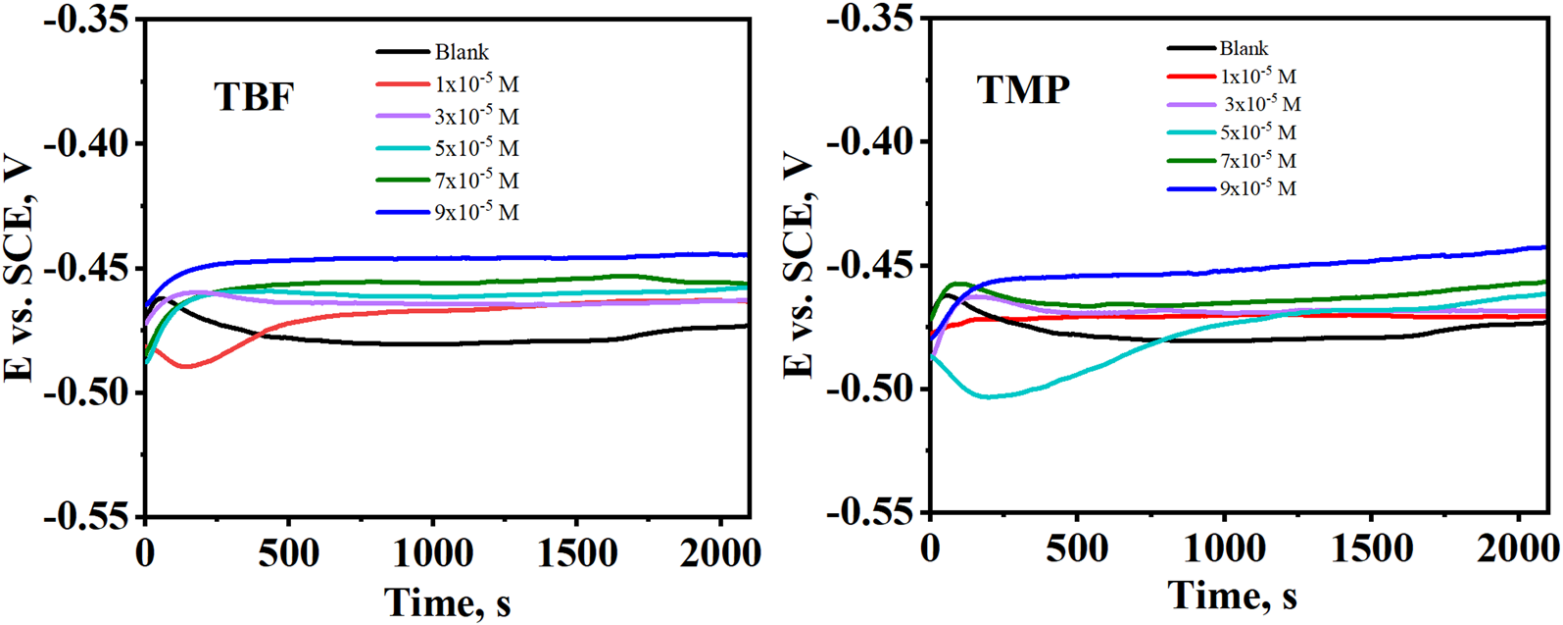
OCP-time plots for carbon steel in the uninhibited and inhibited media with TBF and TMP at 298 K

The OCP-time curves for carbon steel submerged in 0.5 M H_2_SO_4_ with and without the addition of tested inhibitors at several concentrations at 298 K are presented in Fig. 1. In each instance, the inhibitors’ adsorption onto the carbon steel causes the initial potential to gradually shift towards higher positive values (more anodic values) before stabilizing. The anodic shift shows that the inhibitors have adhered well to the carbon steel, impeding the anodic oxidation reaction [26, 27]. The shift is less noticeable at low concentrations, although it remains greater than that of the control without inhibitors. These findings verify that TBF and TMP enhance the resistance of carbon steel to corrosion in acidic conditions, with increased efficacy at higher concentrations [28]. Additionally, there were very slight changes in potential after 35 min of immersion. This suggests that equilibrium is attained after this period, enabling the start of electrochemical measurements in stable conditions [4, 29].

### PP tests

**Fig. 2:**
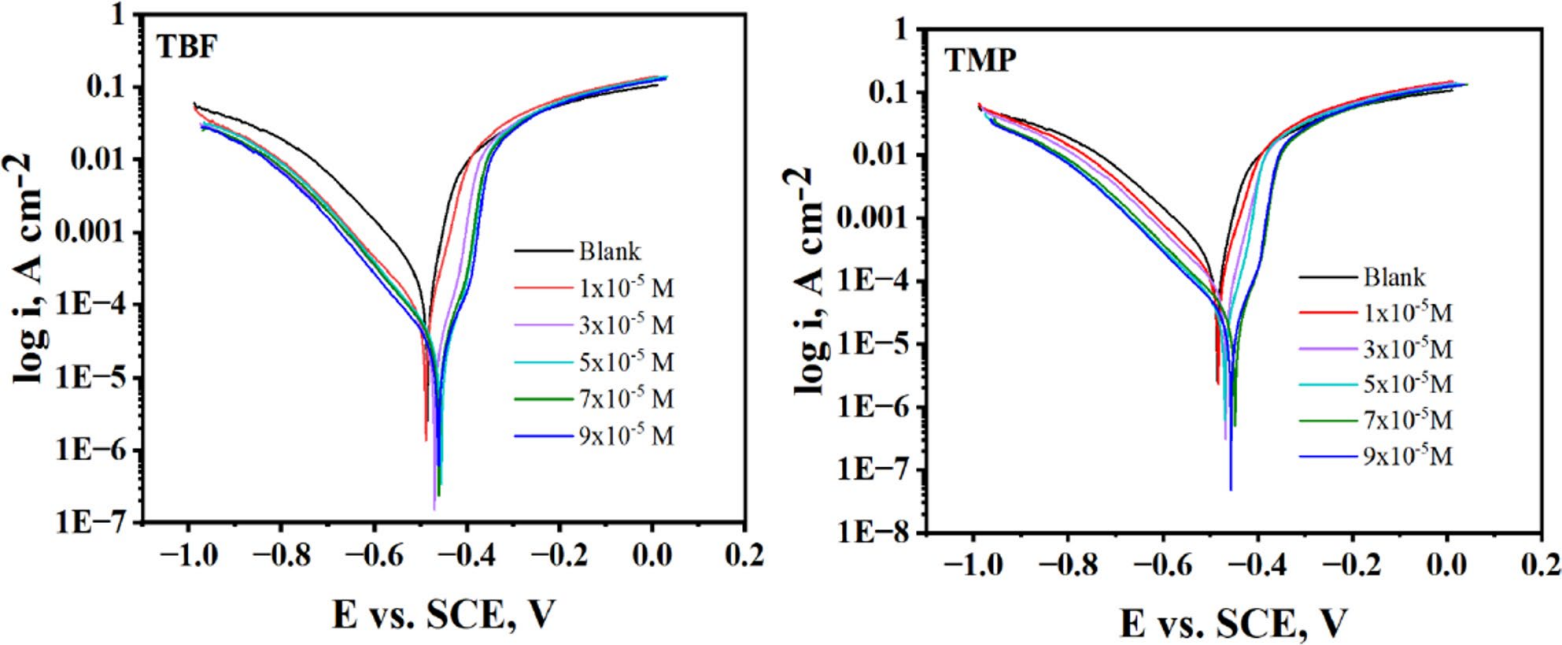
Tafel curves for carbon steel in 0.5 M H_2_SO_4_ without and in the presence of different doses of TBF and TMP at 298 K

Tafel polarization plots were studied at 298 K, as appeared in Fig. 2, and the computed parameters are collected in Table 2. Equation (2) was employed to compute the %IE [30].

2 $${\%}\mathrm{I}\mathrm{E}=\frac{{i}_{corr}^{^\circ\:}-{i}_{corr}}{{i}_{corr}^{^\circ\:}}\times\:100$$

where the current density values in the absence and presence of the inhibitor are denoted by $\:{i}_{corr}^{^\circ\:}$ and $\:{i}_{corr}$, respectively. Equations (3) and (4) were applied to compute *R*_p_ and corrosion rate (CR), respectively [31, 32].

3 $$ R_{p} = \,\frac{{\beta \,_{{\mathrm{a}}} \beta \,_{c} }}{{2.303(\beta \,_{{\mathrm{a}}} + \beta \,_{c} )i_{{{\mathrm{corr}}}} }} $$

4 $$\:{\mathrm{C}\mathrm{R}}_{\mathrm{m}\mathrm{p}\mathrm{y}}=\frac{129\times\:\mathrm{a}\mathrm{t}\mathrm{o}\mathrm{m}\mathrm{i}\mathrm{c}\:\mathrm{m}\mathrm{a}\mathrm{s}\mathrm{s}\:\left(\mathrm{g}\right)\times\:{i}_{corr\:}\left(\mathrm{m}\mathrm{A}\:{\mathrm{c}\mathrm{m}}^{-2}\right)}{\mathrm{n}\mathrm{u}\mathrm{m}\mathrm{b}\mathrm{e}\mathrm{r}\:\mathrm{o}\mathrm{f}\:\mathrm{e}\mathrm{l}\mathrm{e}\mathrm{c}\mathrm{t}\mathrm{r}\mathrm{o}\mathrm{n}\:\mathrm{t}\mathrm{r}\mathrm{a}\mathrm{n}\mathrm{s}\mathrm{f}\mathrm{e}\mathrm{r}\mathrm{e}\mathrm{d}\times\:\mathrm{d}\mathrm{e}\mathrm{n}\mathrm{s}\mathrm{i}\mathrm{t}\mathrm{y}\:\left(\mathrm{g}{\mathrm{c}\mathrm{m}}^{-3}\right)\:}$$

The linear sections of the Tafel branches were extrapolated to the corresponding *E*_corr_ to get the values of $\:{i}_{corr}$. When related to the blank solution, Table 2 showed that $\:{i}_{corr}$ and CR diminished when the tested compounds were present. Consequently, the inhibitory effectiveness increased significantly, reaching 95.3% and 93.5% for TBF and TMP, respectively, at the optimum concentration, which verifies their adsorption and slows down the dissolution [32, 33]. However, the values of *E*_corr_ were not considerably altered by the addition of the investigated inhibitors, indicating that these substances do not appreciably alter the metal surface potential. The information found in the literature suggests that an inhibitor is considered mixed when the change in corrosion potential is less than 85 mV [34]. This type of inhibitor offers a balanced effect against both kinds of electrochemical reactions by influencing both the cathodic and anodic processes. The experimental findings and this observation imply that both TBF and TMP function as mixed-type inhibitors (roughly 36 mV). They help to lower the overall rate of corrosion by concurrently affecting the anodic and cathodic reactions [35]. The addition of TBF and TMP does not significantly alter the *β*_a_ and *β*_c_ values, indicating that the inhibitory mechanism remains unchanged [36]. Increasing *R*_p_ values is attributed to TBF and TMP’s adsorption through the dissolution of carbon steel, due to their blocking of active centers [37]. There is consistency in the trend of change between R_p_ values and the R_p_ values derived by EIS. As a result, the polarization resistances obtained from both electrochemical approaches are in good agreement.

**Table 2:** Tafel parameters of TBF and TMP at 298 K

Inhibitors	Conc., (x10^− 5^ M)	i_corr_., µA.cm^− 2^± SD	-E_corr_, mV vs. SCE	β_a_, mVdec^− 1^	-β_c_, mVdec^− 1^	*R*_*p*_, Ω.cm²	CR, mpy	θ	%IE
Blank	---	324.0 ± 4.08	486.0	77.10	174.9	71.71	147.8	–	–
TBF	1	54.70 ± 3.92	477.0	40.90	80.80	215.56	24.98	0.831	83.1
	3	24.30 ± 1.39	462.0	43.20	116.9	563.65	11.08	0.925	92.5
	5	17.50 ± 1.71	459.0	42.80	108.2	760.96	7.975	0.946	94.6
	7	16.10 ± 2.62	464.0	42.60	93.80	790.09	7.349	0.950	95.0
	9	15.30 ± 1.31	465.0	53.50	84.90	931.41	7.008	0.953	95.3
TMP	1	99.80 ± 2.21	476.0	43.40	93.20	128.83	45.57	0.692	69.2
	3	60.80 ± 1.98	461.0	39.20	129.5	214.90	27.79	0.812	81.2
	5	29.30 ± 2.69	450.0	42.70	125.3	471.96	13.37	0.910	91.0
	7	22.20 ± 2.20	455.0	49.80	120.9	689.88	10.13	0.931	93.1
	9	21.20 ± 1.80	455.0	56.50	125.1	797.19	9.662	0.935	93.5

### EIS tests

**Fig. 3:**
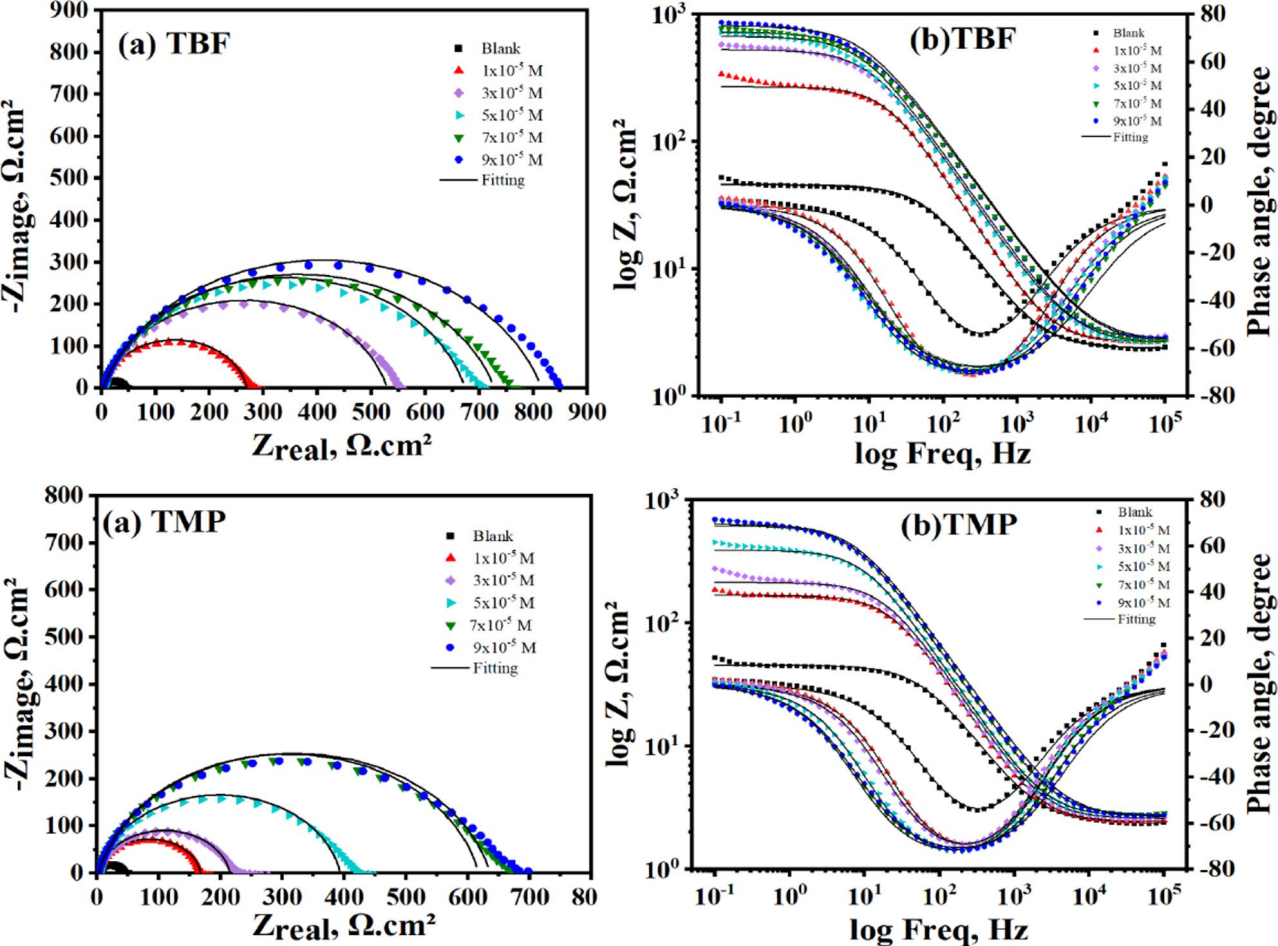
Nyquist (**a**) and Bode (**b**) diagrams for carbon steel in uninhibited and inhibited solutions with different concentrations of TBF and TMP at 298 K

**Fig. 4:**
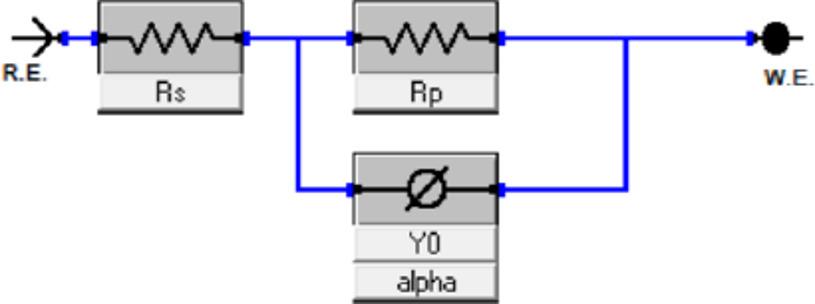
The circuit model for EIS fitting

To precisely ascertain the inhibitory efficiency, EIS tests were conducted as a supplemental and independent method. Additionally, EIS made it possible to determine the inhibition mechanism as well as the kinetic parameters of the electrochemical process [38]. Figure 3a and b display the Nyquist and Bode plots of carbon steel before and after the addition of various doses of tested inhibitors at 298 K, respectively. Capacitive depression semicircles in Fig. 3a indicate that the charge transfer is the cause of carbon steel corrosion. The surface roughness and inhomogeneities of the carbon steel could be the cause of the depressed properties. The diameter of the semicircles grows with increasing inhibitor concentration, which lowers the dissolution rate [39]. Furthermore, neither TBF nor TMP was found to have an evident effect on the Nyquist diagrams in either the inhibited or uninhibited systems, indicating that they did not affect the mechanism of the corrosion reaction [40]. A suitable equivalent circuit was chosen to simulate the experimental data, as displayed in Fig. 4. This model effectively simulates the electrochemical behavior of the system, confirming its suitability for the present investigation [41]. The quality of the fitting was evaluated based on the goodness-of-fit values, which demonstrated an excellent agreement with the proposed equivalent circuit. The impedance parameters are estimated and recorded in Table 3 using the fitted circuit. Equation (5) can be employed to compute the %IE [42].

5 $${\%}\mathrm{I}\mathrm{E}=\frac{{R}_{ct}-{R}_{ct}^{^\circ\:}}{{R}_{ct}}\times\:100$$

where the charge transfer resistances without and with inhibitor are denoted by $\:{R}_{ct}^{^\circ\:}\:$and $\:{R}_{ct}$, respectively. Bode diagrams for the carbon steel following exposure to 0.5 M H_2_SO_4_, both with and without different doses of TBF and TMP, are shown in Fig. 3b. The log Z values appear to sharply increase with increasing inhibitor concentration. This suggests that increased inhibitor concentrations improve the adsorbed protective layer. The presence of a single time constant was shown in the Bode plots for TBF and TMP. Moreover, the Bode phase plot’s significant phase angle (ϴ) values verify that higher inhibitory concentration results in more inhibitory behavior [19, 43, 44]. Based on the data in Table 3, it was noted that, in comparison to the blank system (*R*_ct_= 41.90 Ω.cm^2^), the values of *R*_ct_ grew progressively as the inhibitor concentration peaked to maximum values of 812.6 and 633.2 Ω.cm^2^ for TBF and TMP, respectively. Furthermore, Table 3 demonstrates that as the dose of inhibitor rose, inhibition efficiency increased while double-layer capacitance (C_dl_) values declined. The decreased C_dl_ values are produced by the addition of inhibitors, most likely because of inhibitor scaffolds at the metallic surface replacing water molecules. According to the Helmholtz model, the inhibitor may also decrease capacitance by thickening the double layer [45]. The strong adsorption of TBF and TMP is indicated by the increase and decrease in *R*_ct_ and C_dl_ values, respectively, forming a protective layer that hinders corrosive species from reaching the metal surface. Instead of using C_dl_, the constant phase element (CPE) is usually employed for a precise and accurate fit. Equation 6 links C_dl_ to the CPE constants (n and Y_o_) [46].

6 $$ C_{{dl}} = \left[ {Y_{0} R_{{et}} ^{{\left( {1 - n} \right)}} } \right]^{{{\raise0.5ex\hbox{$\scriptstyle 1$} \kern-0.1em/\kern-0.15em \lower0.25ex\hbox{$\scriptstyle n$}}}} $$

where n describes the CPE exponent and $\:{\mathrm{Y}}_{0}$ denotes the CPE magnitude. The n values range from 0 to 1, with a value close to unity indicating a smoother metal surface [47].

**Table 3:** EIS parameters for carbon steel in uninhibited and inhibited solutions with different concentrations of TBF and TMP at 298 K

Inhibitors	Conc., (x10^− 5^ M)	*R*_s_, Ω.cm²	*R*_ct,_ Ω.cm² ± SD	Y_o_x10^−6^, µΩ^−1^s^*n*^cm^−2^	*n*	C_dl_, µF/cm²	Goodness of Fit	θ	%IE
Blank	---	2.379	41.90 ± 2.04	134.9	0.873	63.5	0.530	–	–
TBF	1	2.660	266.2 ± 1.76	52.54	0.908	34.1	0.511	0.843	84.3
	3	2.844	525.7 ± 0.65	51.67	0.858	28.5	0.330	0.920	92.0
	5	2.590	670.6 ± 1.14	50.93	0.850	28.0	0.378	0.938	93.8
	7	2.602	725.4 ± 0.45	47.90	0.818	22.7	0.391	0.942	94.2
	9	2.697	812.6 ± 0.61	45.38	0.820	22.0	0.453	0.948	94.8
TMP	1	2.452	165.5 ± 1.27	66.47	0.914	43.4	0.384	0.747	74.7
	3	2.578	212.5 ± 1.51	65.20	0.900	40.5	0.501	0.803	80.3
	5	2.712	390.7 ± 1.76	61.35	0.897	40.0	0.377	0.893	89.3
	7	2.740	614.7 ± 1.02	57.22	0.877	35.8	0.388	0.932	93.2
	9	2.649	633.2 ± 1.84	55.72	0.863	32.7	0.422	0.934	93.4

### Effect of temperature

**Fig. 5:**
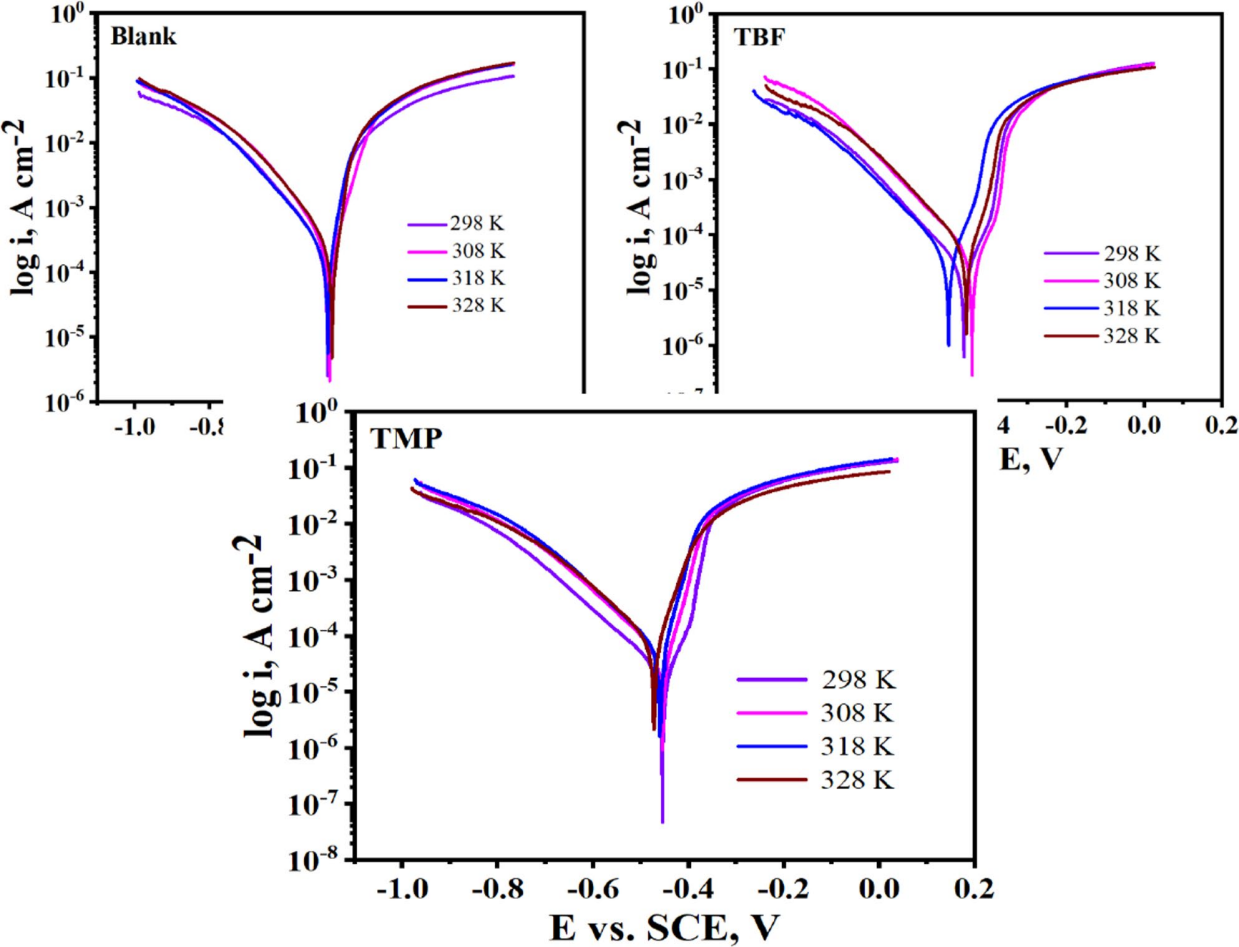
PP measurements at various temperatures before and after the addition of the optimum dose of TBF and TMP

**Fig. 6:**
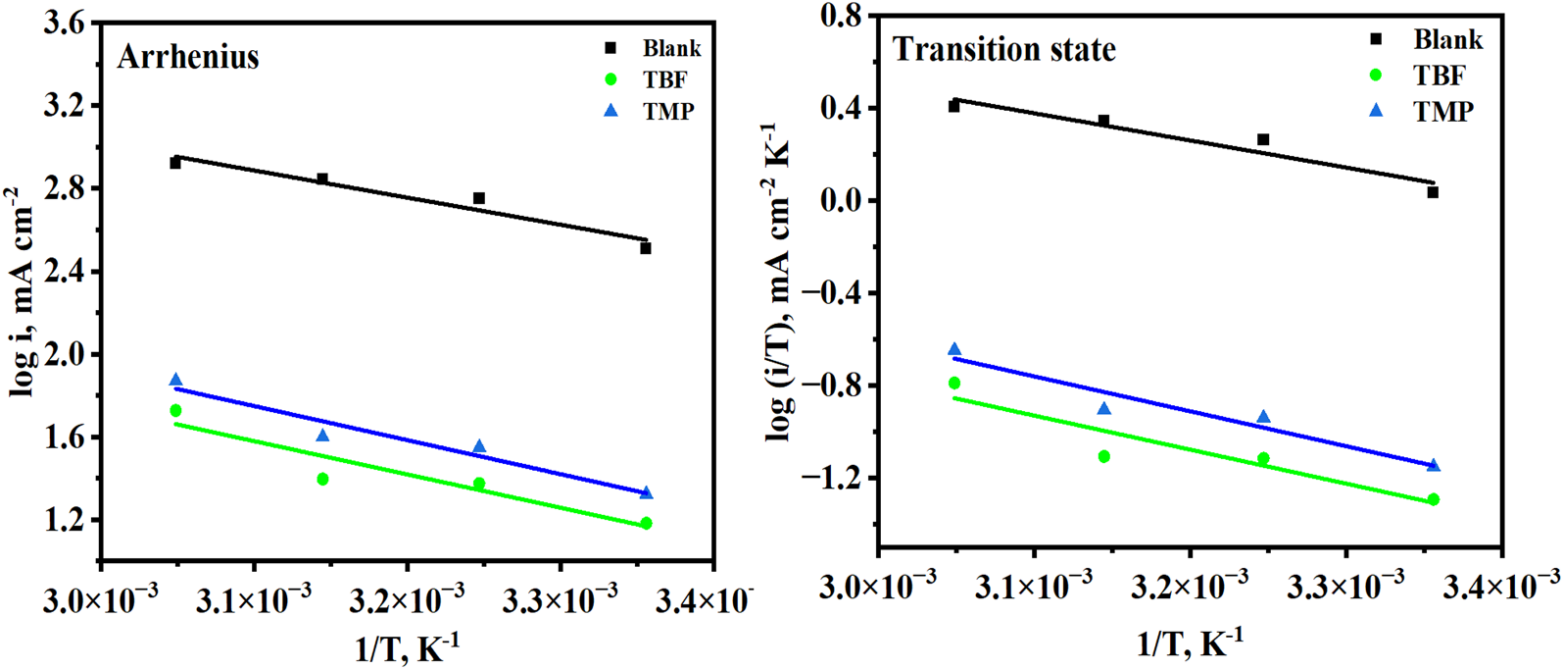
Arrhenius and transition state plots for carbon steel in uninhibited and inhibited solutions with the optimum dose of TBF and TMP

**Fig. 7:**
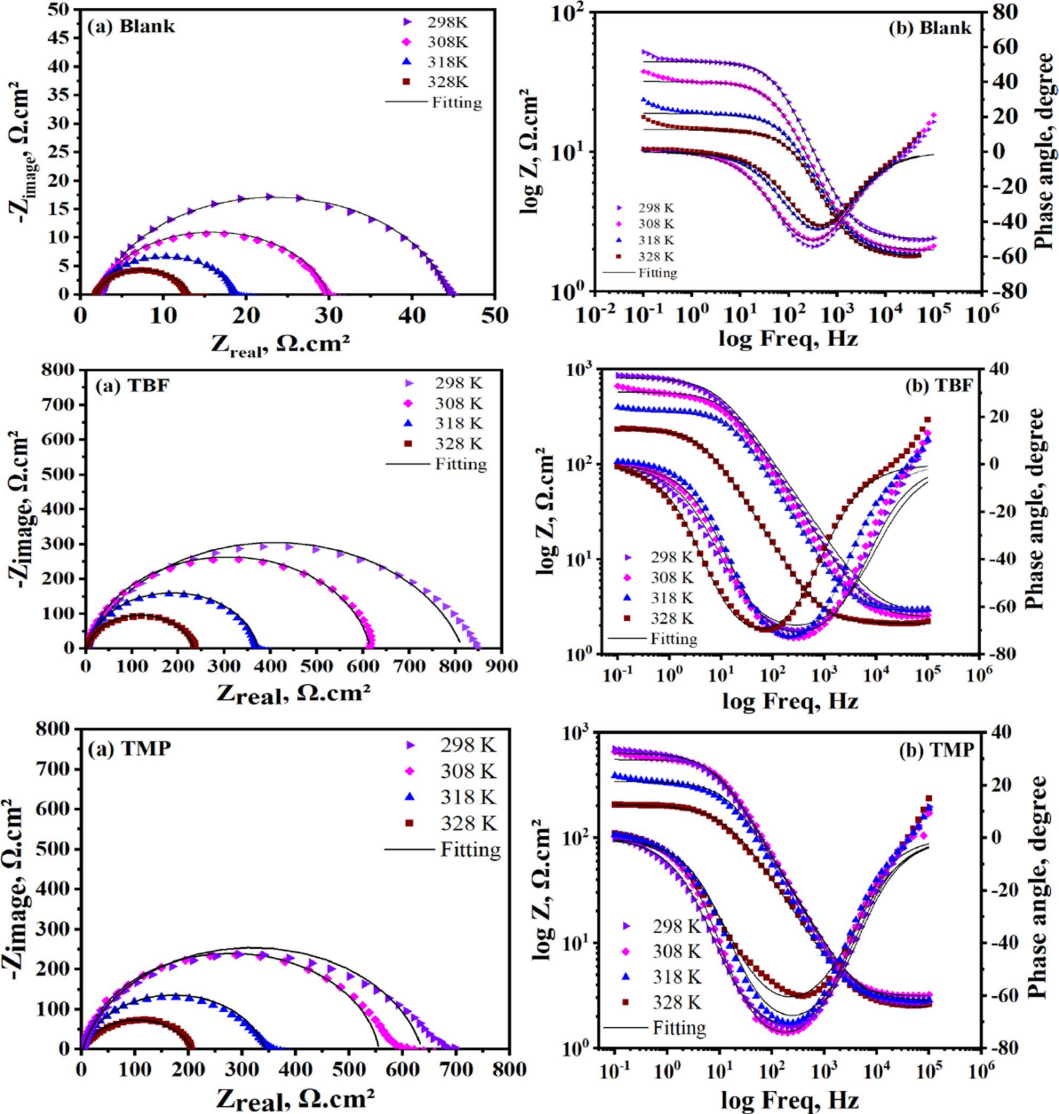
Nyquist (**a**) and Bode (**b**) curves for carbon steel in 0.5 M H_2_SO_4_ at various temperatures, both with and without the optimum dose of TBF and TMP

Temperature changes can drastically alter the behavior of a material vulnerable to a corrosive environment. In this instance, it influences the interaction between the inhibitor and the metal, which may accelerate dissolution and result in desorption [48]. We assessed the impact of this parameter on the effectiveness of inhibitors in the range of 298–328 K using PP and EIS tests to ascertain the durability of the interface. Figure 5 shows the results of plotting the PP measurements in 0.5 M H_2_SO_4_ before and with the optimum dose of TBF and TMP at various temperatures. Table 4 displays their extracted and computed data, which revealed that the *i*_corr_. values increase as T increases, and this effect is more pronounced for the non-inhibited solution [49, 50]. Additionally, for the optimal concentration, we have seen a slight increase in inhibition efficiency as T increases from 298 K to 318 K and then decreases slightly at 328 K, indicating that higher temperatures make the equilibrium of adsorption-desorption shift towards desorption, which results in a slight decrease in the effectiveness of TBF and TMP [51]. This certifies the constancy of the adsorbed layer at higher temperatures. It is possible to calculate activation energy ($\:{\mathrm{E}}_{\mathrm{a}}^{\mathrm{*}}$), activation enthalpy (ΔH*), and activation entropy (ΔS*) through analyzing the Tafel data. For this, Fig. 6 was displayed. Logarithmic formulations of the Arrhenius and the transition state equations are validated by the created graphs, which show linear relationships [52]:

7 $$\:{\mathrm{i}}_{\mathrm{c}\mathrm{o}\mathrm{r}\mathrm{r}}=\mathrm{A}\:\mathrm{e}\mathrm{x}\mathrm{p}\left(\frac{{-\mathrm{E}}_{\mathrm{a}}^{\mathrm{*}}\:}{\mathrm{R}\mathrm{T}}\right),$$

8 $$\:{\mathrm{i}}_{\mathrm{c}\mathrm{o}\mathrm{r}\mathrm{r}}=\frac{\mathrm{R}\mathrm{T}}{\mathrm{N}\mathrm{h}}\mathrm{e}\mathrm{x}\mathrm{p}\left(\frac{{\triangle\mathrm{S}}^{\mathrm{*}}}{\mathrm{R}}\right)\mathrm{e}\mathrm{x}\mathrm{p}\left(\frac{-{\triangle\mathrm{H}}^{\mathrm{*}}}{\mathrm{R}\mathrm{T}}\right),$$

where R is the gas constant (J K^− 1^ mol^− 1^), A stands for the pre-exponential factor, and T is the absolute temperature (K). $\:{\mathrm{E}}_{\mathrm{a}}^{\mathrm{*}}$ was computed using the slope value of the Arrhenius plot (log i_corr_ vs. (1 /T)) in Fig. 6, as shown in Table 5 [53]. Using the slope = -ΔH* / (2.303R) and intercept = [log(R/Nh) + ΔS* / (2.303R)], the values of ΔH* and ΔS* were computed from the Transition plot (log(i_corr_/T) vs. (1 /T)) displayed in Fig. 6 [54]. Table 5 lists the$\:{\:\mathrm{E}}_{\mathrm{a}}^{\mathrm{*}}$ values, which appear to remain constant in the presence of TBF and TMP. This demonstrates that TBF and TMP adsorption on the carbon steel surface is nearly a type of chemical adsorption. The fact that the $\:{\:\mathrm{E}}_{\mathrm{a}}^{\mathrm{*}}$ for TBF to be less than that of TMP indicates that TBF is chemically adsorbed on the surface [55]. The positive values of ΔH* in Table 5 suggest that the dissolution of carbon steel is endothermic and challenging [56]. The activation of the complex, not the dissociation, was the step that determined the rate, as indicated by the negative sign of ΔS*. Because of the increased order, the inhibition efficiency (from reactant to activated complex) increased [55]. The outcomes obtained from EIS were in line with previous findings, which are displayed in Fig. 7a and b, and the related parameters are tabulated in Table 6. The R_ct_ values decrease with temperature. TBF and TMP significantly reduced the dissolutioan response of carbon steel dueto the higher R_ct_ values observed in their presence compared to those obtained without them. The Nyquist plots at different temperatures are semicircular and get smaller in diameter as the temperature rises. Increasing temperatures did not affect the appearance of the Nyquist spectra. This demonstrated that, even after introducing TBF and TMP, the mass loss-controlled mechanism of the carbon steel corrosion reaction was unaffected by temperature [57]. For the three systems under investigation, the values of impedance modulus drop at lower frequencies as the temperature rises, as indicated in Bode plots in Fig. 7b [58].

**Table 4:** The PP-derived values in 0.5 M H_2_SO_4_, both without and with adding the optimum dose of TBF and TMP at different temperatures

Temp, K	Compound	-E_corr,_ mV vs. SCE	i_corr,_ µAcm^− 2^ ± SD	C.*R*. mpy		θ	%IE
298	Blank	486.0	324.0 ± 4.08	147.8		–	–
	TBF	465.0	15.30 ± 1.31	7.01		0.953	95.3
	TMP	455.0	21.20 ± 1.80	9.66		0.935	93.5
308	Blank	485.0	566.0 ± 1.63	258.4		–	–
	TBF	473.0	23.80 ± 3.10	10.87		0.958	95.8
	TMP	465.0	35.60 ± 3.67	16.24		0.937	93.7
318	Blank	479.0	705.0 ± 4.08	327.7		–	–
	TBF	473.0	25.00 ± 2.45	11.42		0.965	96.5
	TMP	466.0	39.90 ± 3.51	18.22		0.943	94.3
328	Blank	469.0	836.0 ± 4.45	381.8		–	–
	TBF	464.0	53.60 ± 4.33	24.50		0.936	93.6
	TMP	471.0	74.50 ± 4.49	34.04		0.911	91.1

**Table 5:** Computed activation parameters at different temperatures

Compounds	Arrhenius		Transition state			
	Slope	$\:{\mathrm{E}}_{\mathrm{a}}^{\mathrm{*}},$ kJ mol^− 1^	Slope	Intercept	ΔH^*^, kJ mol^− 1^	-ΔS^*^, J mol^− 1^ K^− 1^
Blank	– 1310.13	25.09	– 1174.44	4.02017	22.49	120.59
TBF	– 1608.05	30.79	– 1472.36	3.63686	28.19	127.93
TMP	– 1645.28	31.50	– 1509.59	3.92159	28.90	122.48

**Table 6:** EIS parameters in 0.5 M H_2_SO_4_ before and after the addition of the optimum dose of TBF and TMP at 298–328 K

Temp, K	Compound	*R*_s,_ Ω.cm²	*R*_ct,_ Ω.cm² ± SD	Goodness of fit	Θ	%IE
298	Blank	2.379	41.90 ± 2.04	0.530	–	–
	TBF	2.697	812.6 ± 0.61	0.453	0.948	94.8
	TMP	2.649	633.2 ± 1.84	0.422	0.934	93.4
308	Blank	1.971	30.00 ± 2.45	0.777	–	–
	TBF	2.607	613.0 ± 4.08	0.289	0.951	95.1
	TMP	3.146	553.0 ± 3.27	0.230	0.946	94.6
318	Blank	1.844	17.00 ± 4.90	0.329	–	–
	TBF	2.964	368.7 ± 3.47	0.227	0.954	95.4
	TMP	2.847	340.0 ± 5.72	0.359	0.950	95.0
328	Blank	1.784	12.60 ± 0.90	0.375	–	–
	TBF	2.109	231.6 ± 3.80	0.549	0.946	94.6
	TMP	2.407	203.9 ± 4.82	0.692	0.938	93.8

### Adsorption isotherm

**Fig. 8:**
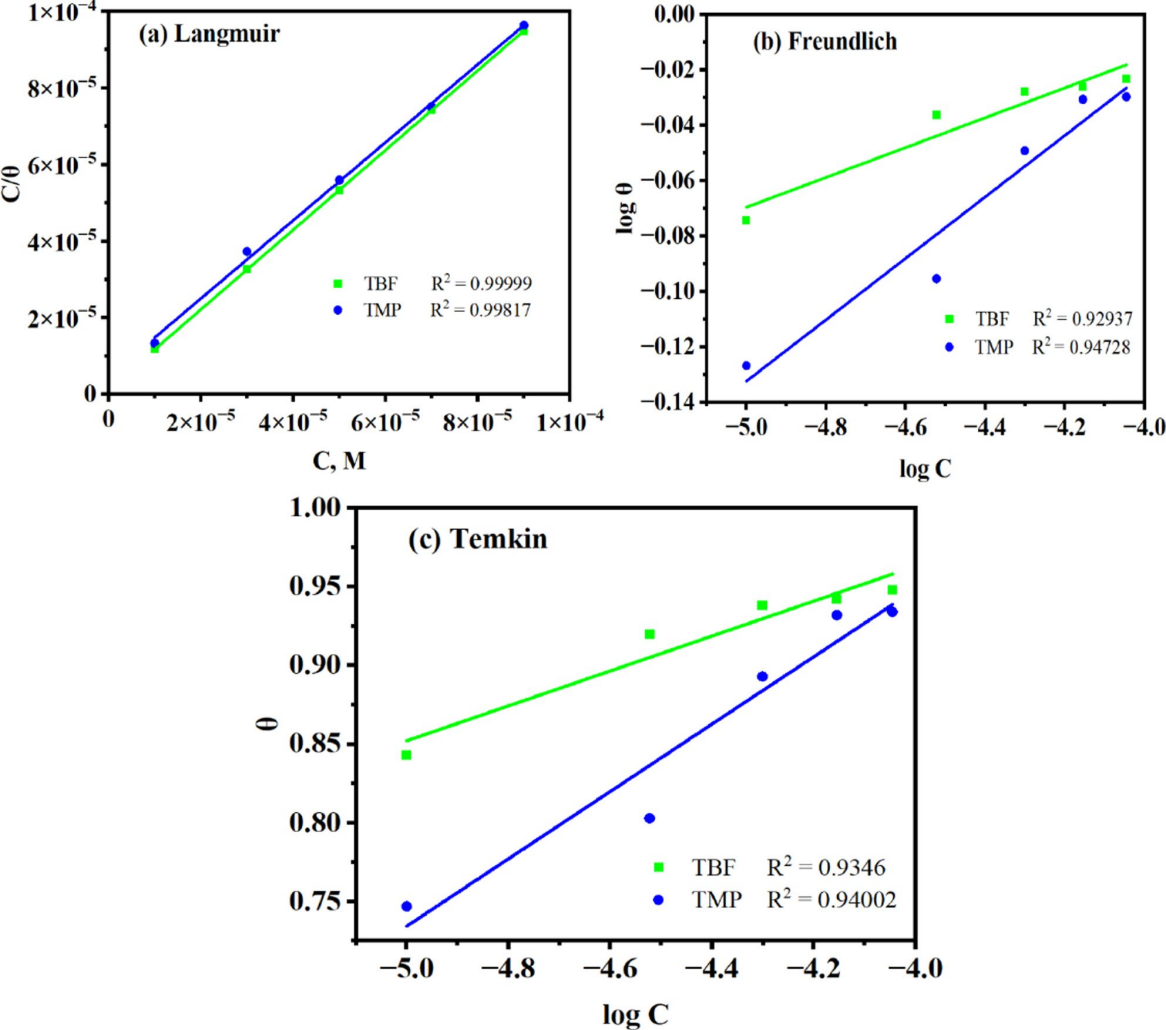
The tested adsorption isotherms at 298 K in 0.5 M H_2_SO_4_ containing TBF and TMP

Using well-known adsorption isotherms, such as the Langmuir, Freundlich, and Temkin models, the adsorption behavior was studied via EIS data (Fig. 8). In contrast to the Freundlich and Temkin models, the Langmuir model is the most appropriate for fitting the EIS data, based on the linear regression analysis (R^2^) of nearly unity, which studied *via* the following formula [56, 59]:

9 $$\:\frac{C}{{\uptheta\:}}=\frac{1}{{\mathrm{K}}_{\mathrm{a}\mathrm{d}\mathrm{s}}}+\mathrm{C}$$

In this case, C stands for inhibitor concentration, θ is the surface coverage area occupied by inhibitor molecules, and K_ads_ for adsorption isotherm constant. Figure 8 illustrates how Eq. 9 is represented as C/θ against C. K_ads_ values were estimated from the intercepts and connected to the Gibbs energy of adsorption ($\triangle\:{\mathrm{G}}_{\mathrm{a}\mathrm{d}\mathrm{s}}^{^\circ\:}$) as follows [60, 61]:

10 $$\triangle\:{\mathrm{G}}_{\mathrm{a}\mathrm{d}\mathrm{s}}^{^\circ\:}=-\mathrm{R}\mathrm{T}\mathrm{l}\mathrm{n}\left({\mathrm{K}}_{\mathrm{a}\mathrm{d}\mathrm{s}}\mathrm{x}55.5\right)$$

where 55.5 is the concentration of water in solution (M). Table 7 lists the computed adsorption parameters. Based on Table 7, the degree of adsorption increases with the value of K_ads_. For both TBF and TMP, the adsorption process’s spontaneity is supported with the negative values of $\triangle\:{\mathrm{G}}_{\mathrm{a}\mathrm{d}\mathrm{s}}^{^\circ\:}$ [62]. When analyzing the type of inhibitor adsorption on metal, physisorption, chemisorption, or mixed, the calculated value of $\triangle\:{\mathrm{G}}_{\mathrm{a}\mathrm{d}\mathrm{s}}^{^\circ\:}$ is essential. According to many studies in the literature, a value of $\triangle\:{\mathrm{G}}_{\mathrm{a}\mathrm{d}\mathrm{s}}^{^\circ\:}$ near − 20 kJmol^− 1^ or less negative suggests that adsorption occurs mainly through electrostatic attraction among charged molecules and surface, a phenomenon referred to as physisorption. Conversely, when $\triangle\:{\mathrm{G}}_{\mathrm{a}\mathrm{d}\mathrm{s}}^{^\circ\:}$ is around − 40 kJmol^− 1^ or more negative, it reveals charge transfer or sharing from the organic scaffolds to the metal, forming coordinate bonds characteristic of chemisorption. The inhibitor exhibits mixed-type adsorption if the value of $\triangle\:{\mathrm{G}}_{\mathrm{a}\mathrm{d}\mathrm{s}}^{^\circ\:}$ falls between − 40 and − 20 kJmol^− 1^ [45, 63]. Coordination bonds were formed when TBF and TMP adsorbed and shared electrons with Fe, as indicated by the computed values of $\triangle\:{\mathrm{G}}_{\mathrm{a}\mathrm{d}\mathrm{s}}^{^\circ\:}$ in Table 7 [64].

**Table 7:** Adsorption parameters extracted from the Langmuir model for TBF and TMP at 298 K

Compounds	Slope	*R* ^2^	K_ads_ x10^4^, M^− 1^	-$\triangle\:{\mathbf{G}}_{\mathbf{a}\mathbf{d}\mathbf{s}}^{\mathbf{o}}$, kJ mol^− 1^
TBF	1.03925	0.99999	69.3	43.28
TMP	1.01847	0.99817	21.2	40.34

### Surface studies

#### AFM

**Fig. 9:**
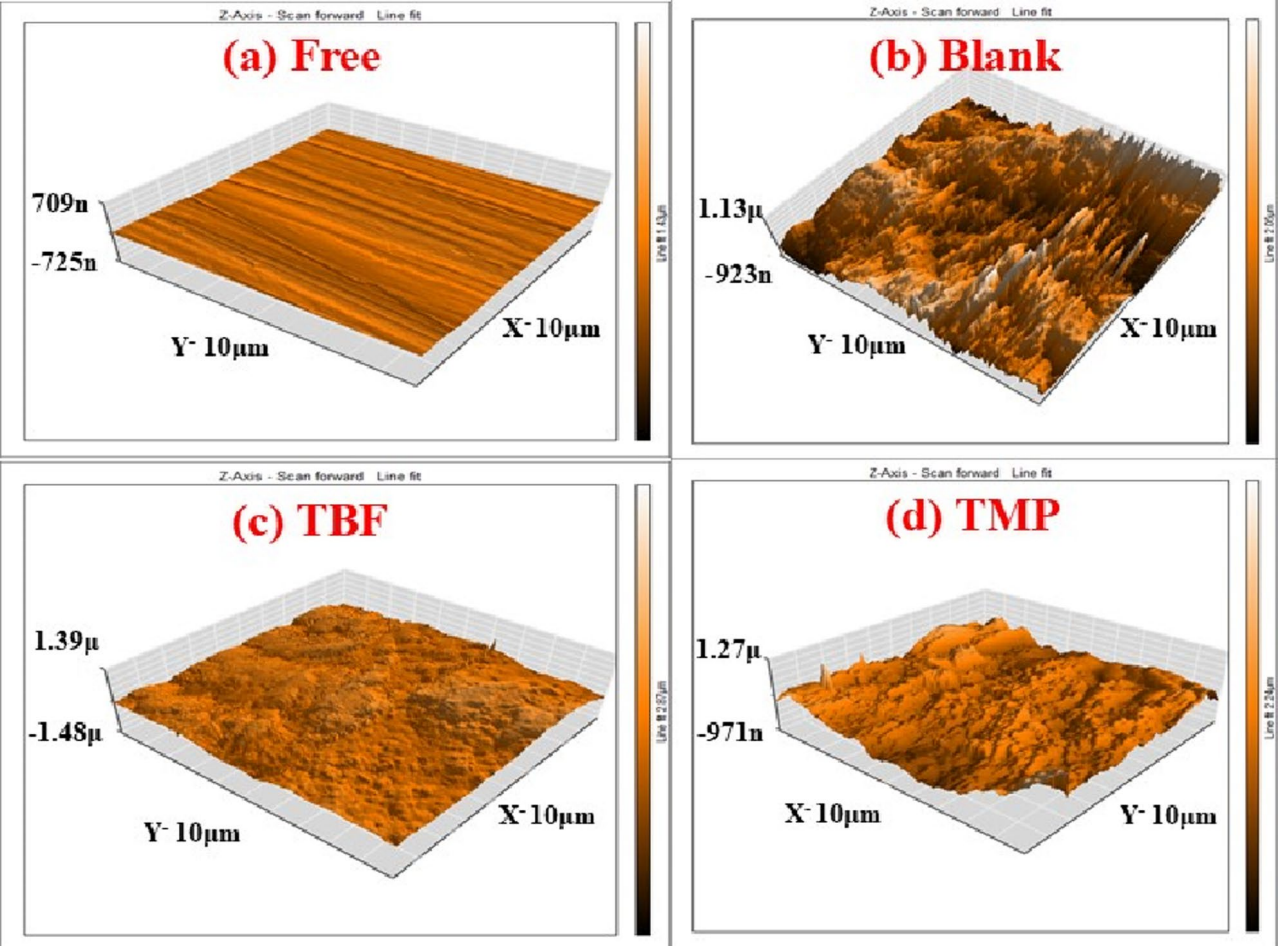
AFM images of a polished sample of carbon steel (**a**), a polished sample in blank solution before (**b**), and following adding the optimal dose of TBF (**c**) and TMP (**d**) for 24 h at 298 K

The roughness of the carbon steel surface was assessed using AFM both with and without the highest dose of TBF and TMP after 24 h of dipping in 0.5 M H_2_SO_4_. Figure 9 shows AFM images of four different samples: (a) carbon steel that has been abraded, (b) metal immersed in blank solution, and (c) and (d) metal immersed in blank with the optimal dose of TBF and TMP, respectively. The surface was smooth with a roughness of 14.946 nm before immersion in blank solution (Fig. 9a). After being submerged in acid (Fig. 9b), the surface degraded and became rougher (761.63 nm); however, the samples treated with the tested inhibitors had smoother surfaces and lower roughness values than the uninhibited sample. Adsorption of TBF (Fig. 9c) and TMP (Fig. 9d) on the surface causes this decrease in roughness, reaching 78.945 nm and 129.18 nm, respectively, by forming strong protective layers that impede metal dissolution [65–67].

#### FT-IR

**Fig. 10:**
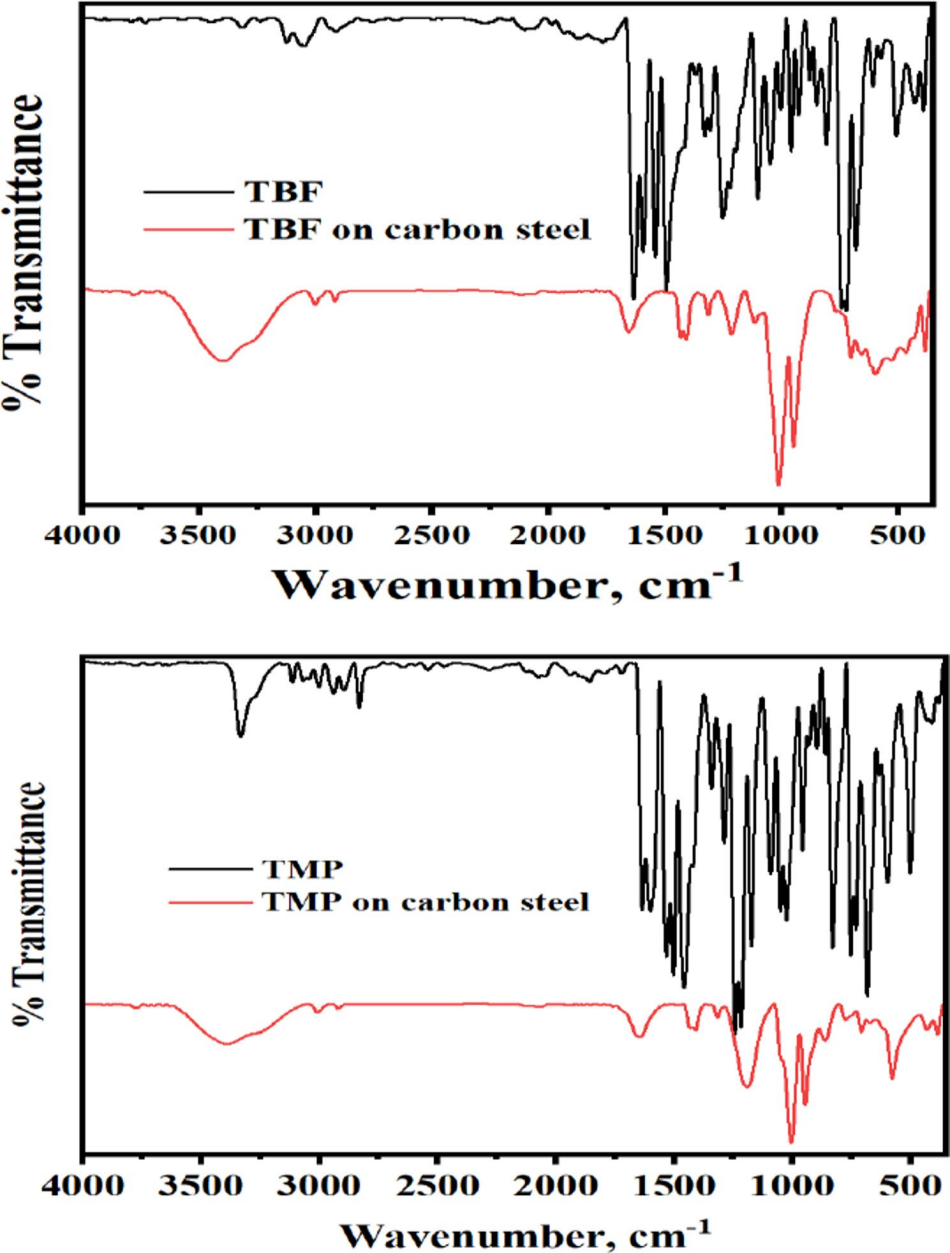
Comparison of the infrared spectra of pure inhibitors and the carbon steel surface at 298 K after 24 h of immersion in 0.5 M H_2_SO_4_ solution containing the optimal dose of the TBF and TMP

FT-IR spectra of the pure solid inhibitors compared with the adsorbed layer of TBF and TMP on carbon steel are shown in Fig. 10 to prove their interaction in 0.5 M H_2_SO_4_. Some peaks between the tested inhibitors’ spectra and the layers adsorbed on the surface shift or vanish, and some peaks also become less prominent. These changes indicate the formation of strong chemical bonds between the inhibitors and the carbon steel surface. This means that chemisorption, which involves bonding at themolecular level, is theprimary mechanism of interaction, while physical adsorption, which is weaker and involves only surface attraction forces, may still occur but is not dominant in this case [68, 69].

#### Contact angle measurements

**Fig. 11:**
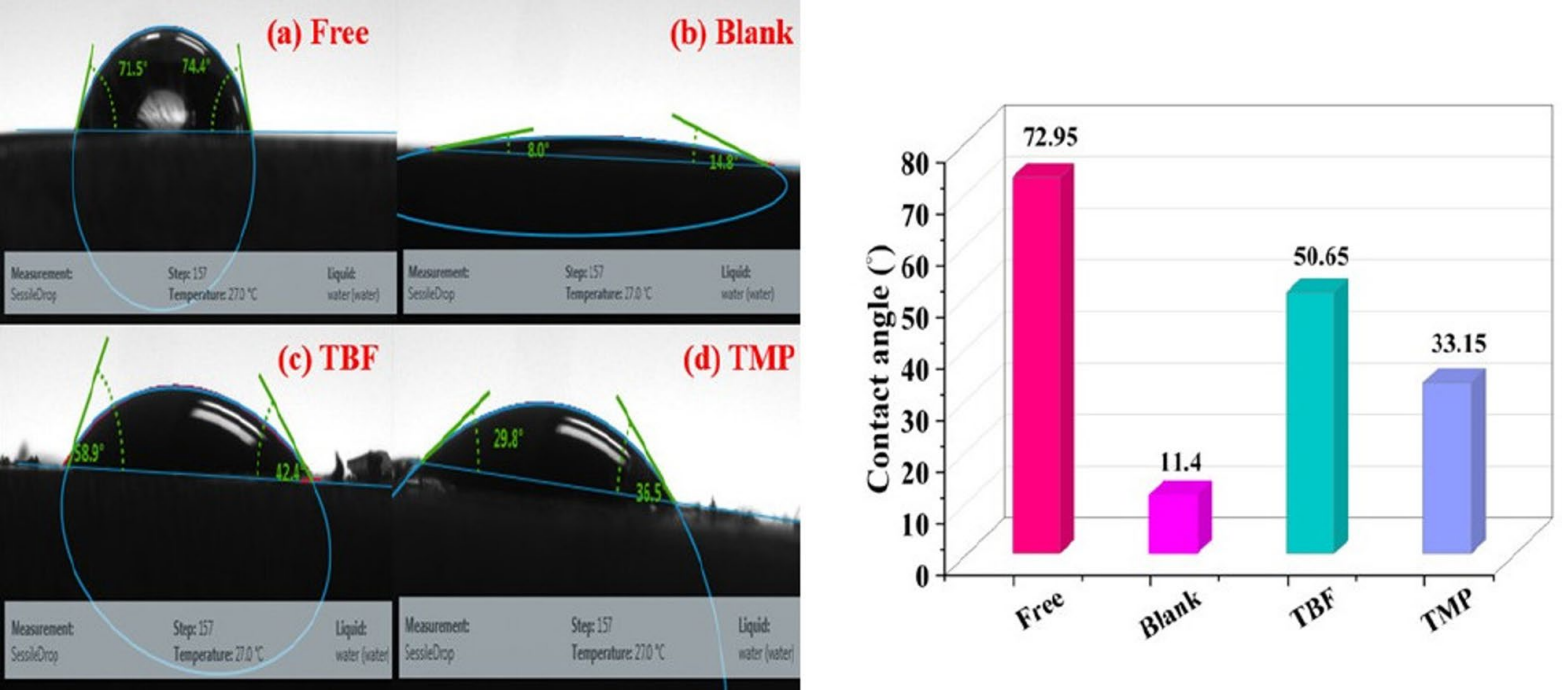
The measured water contact angle on the surface of polished carbon steel (**a**), polished carbon steel in blank solution (**b**), and polished carbon steel in blank solution containing TBF (**c**) and TMP (**d**) inhibitors, respectively, after submersion for 24 h at 298 K

Changes in the hydrophobicity of carbon steel were assessed using the contact angle method, both with and without the investigated inhibitors [70]. A graph of the contact angle of a water droplet on the carbon steel under different conditions is shown in Fig. 11. The polished sample’s water contact angle decreased from 72.95 ° to 11.4° during the corrosion test. The water contact angles for carbon steel protected by TBF and TMP were higher, measuring 50.65° and 33.15°, respectively. The evolution of a protective film of tested inhibitors is suggested by the augmentation in hydrophobicity and diminish in wetting on the surface. Consequently, the surface was protected by synthetic inhibitors, shielding it from corrosive substances. TBF’s higher water contact angle indicated superior surface adsorption compared to TMP [71].

#### XPS

**Fig. 12:**
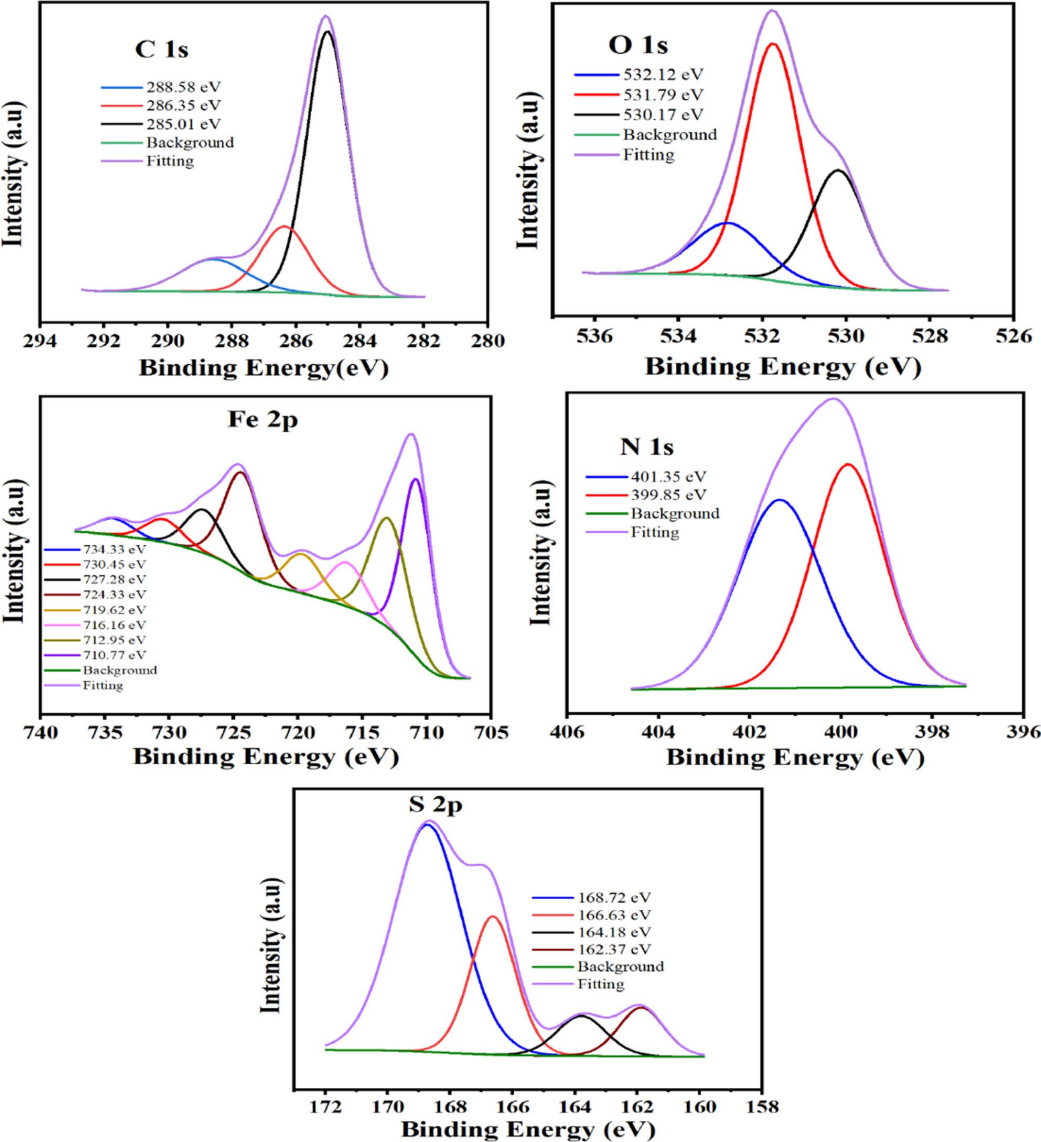
XPS spectra of the adsorbed layer of TBF on the carbon steel

**Fig. 13:**
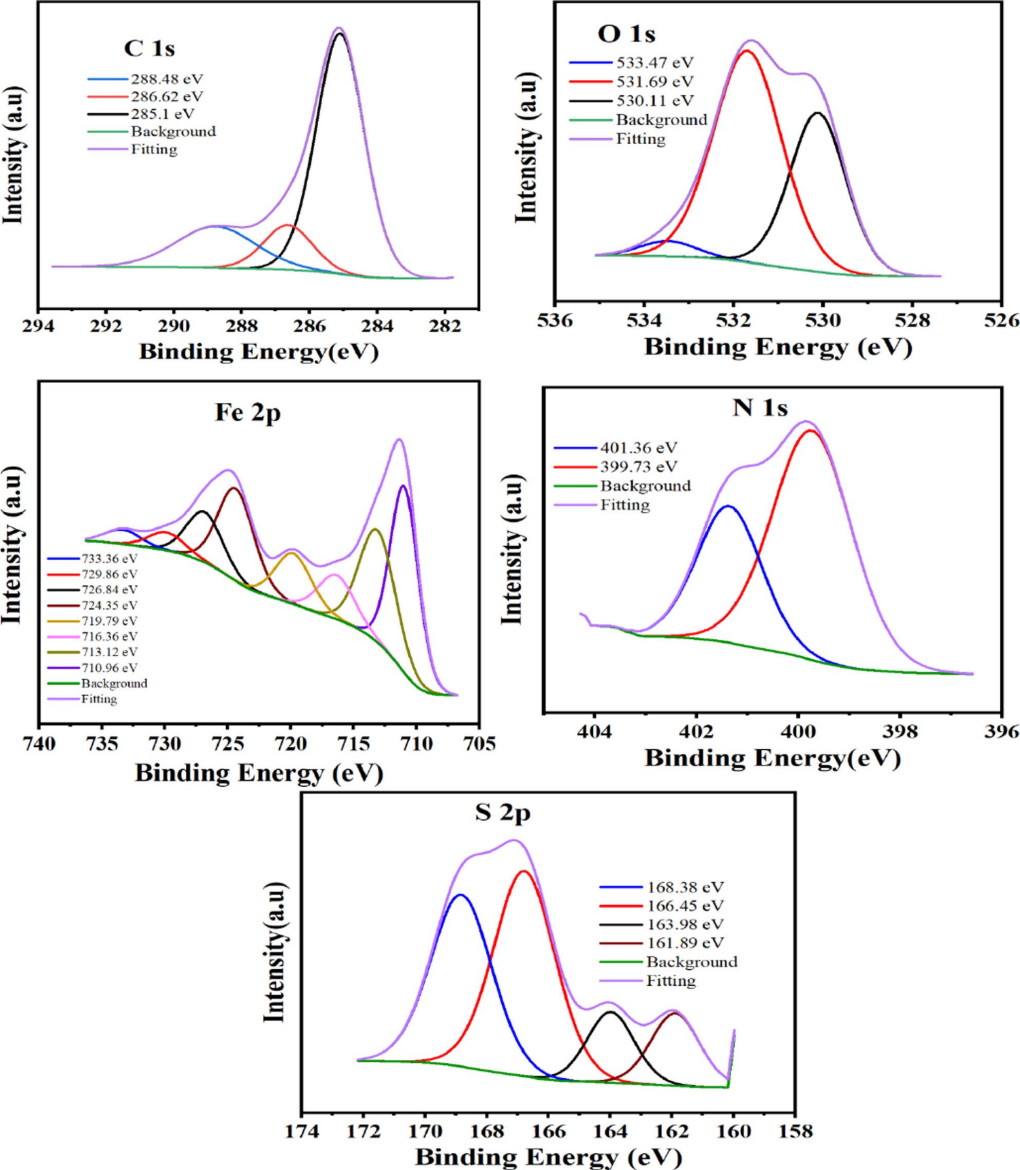
XPS spectra of the adsorbed layer of TMP on the carbon steel

The nature of the adsorbed layer onto the steel is clarified by the XPS study [72]. The carbon steel was submerged in inhibitor solutions in 0.5 M H_2_SO_4_ at the optimum concentration for a full day to produce the XPS spectra as indicated in Figs. 12 and 13 for TBF and TMP, respectively. Three peaks make up the resolved C 1s spectra of carbon steel when TBF and TMP are present. The C–C, C = C, and C–H bonds are shown by the first peak’s appearance at 285.01 and 285.1 eV [73]. The C–O–C and C–N bonds are responsible for the second peak’s appearance at 286.35 and 286.62 eV [1, 74]. The C = N is responsible for the third peak, which appeared at 288.58 and 288.48 eV [75]. In the O 1s spectra, the peaks at 530.17, 530.11 eV and 531.79, 531.69 eV represent iron oxide, or Fe_2_O_3_/Fe_3_O_4,_ and FeOOH, respectively. The water molecules that were deposited showed their peak at 532.12 and 533.47 eV [76]. There are several peaks in the Fe 2p XPS spectra in Figs. 12 and 13 for TBF and TMP, respectively. The peaks observed at 710.77, 710.96 eV are assigned to the Fe 2p_3/2_ (Fe^2+^ oxide state of FeO), while, at 712.95, 713.12 eV, referred to as Fe 2p_3/2_ (Fe^3+^ oxide state of Fe_2_O_3_). The peaks at 716.16, 716.36 eV are attributed to Fe 2p_3/2_ (Fe^2+^ satellite), while at 719.62, 719.79 eV are ascribed to Fe 2p_3/2_ (Fe^3+^ satellite). Fe 2p_1/2_ (Fe^2+^ oxide state of FeO) peaks appeared at 724.33, 724.35 eV, while those for Fe 2p_1/2_ (Fe^3+^ oxide state of Fe_2_O_3_) appeared at 727,28, 726.84 eV. Peaks of Fe 2p_1/2_ (Fe^2+^ satellite) observed at 7*30.45*, 729.86 eV. Additionally, Fe 2p_1/2_ (Fe^3+^ satellite) shows peaks located at 734.33 and 733.36 eV [77]. The XPS spectra of nitrogen (Figs. 12 and 13) displayed two peaks for N-Fe/N-N/N-H and C = N-N at 399.85, 399.73 eV and 401.35, 401.36 eV, respectively [56, 78]. The C-S bond, Fe sulfides, and the Fe-S bond, sulfates are represented by the peaks of S 2p at 162.67, 161.89, 164.18, 163.98, and 166.63, 166.45, 168.72, 168.38 eV, respectively [56, 79]. Overall, the XPS spectra of carbon steel dipped in TBF and TMP acid solutions verified their adsorption, which decreased carbon steel corrosion.

### UV–Visible spectra

**Fig. 14:**
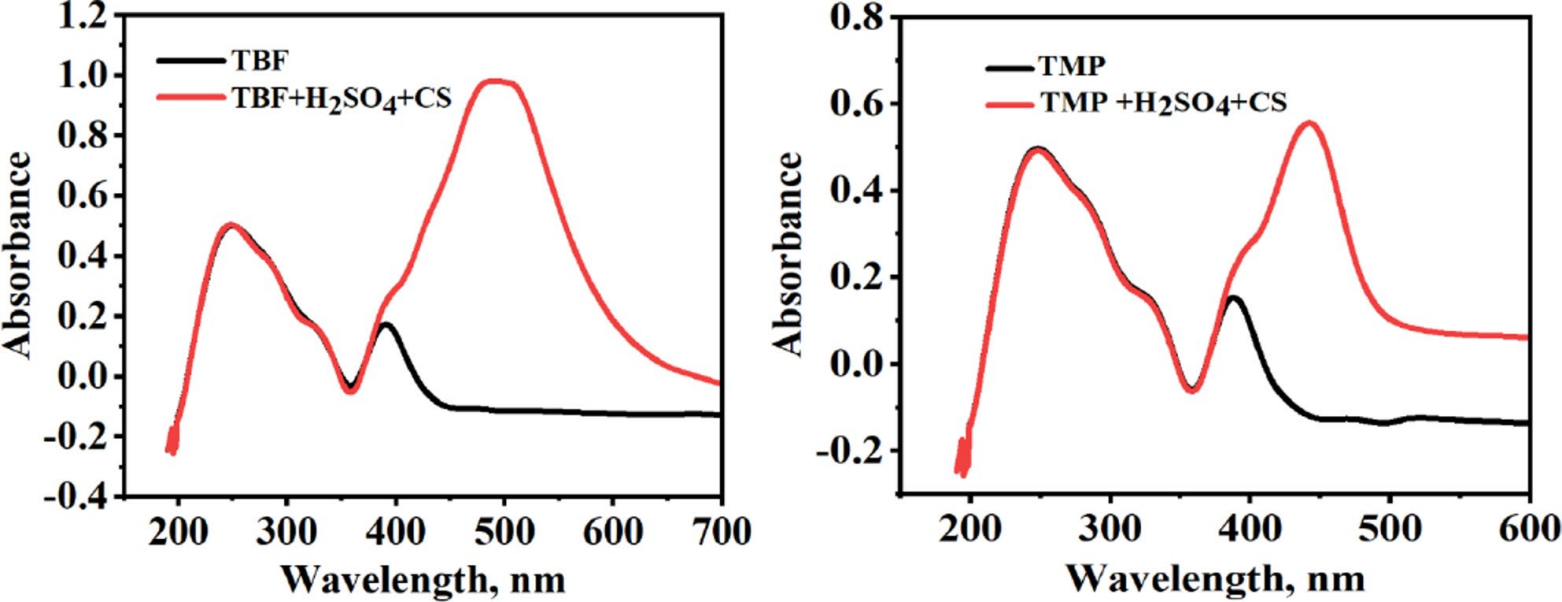
The TBF and TMP UV-Visible spectra both before and after immersion of carbon steel in test solutions for two days at 298 K

The most sensitive technique for directly identifying complex formation is UV-visible spectroscopy, which depends on absorption. Changes in absorbance values or the wavelength of the absorption peak (λ_max_) indicate that a complex is forming between the two species in a solution [80]. Figure 14 illustrates the absorption spectra of TBF and TMP before and after the immersion of carbon steel in the test solutions for two days at 298 K. Initially, TBF and TMP exhibited characteristic absorption peaks at 249 and 391 nm, and at 248 and 388 nm, respectively. These peaks arise from π–π* within the aromatic systems and n–π* transitions involving non-bonding electrons on heteroatoms [81–83]. After carbon steel immersion, noticeable spectral changes were observed, with new peaks appearing at 248 and 490 nm for TBF, and at 248 and 442 nm for TMP. These spectral changes support the assembly of a complex between the Fe^2+^ ions and the investigated inhibitors, validating the inhibition of the metal dissolution process.

### Antibacterial activity

Due to improper or excessivemedication use, the number ofmultidrug-resistant pathogenic bacteria has significantly increased. As a result, it has contributed to both the corrosion caused by microbes and the increase in resistance to antibiotics. The excretions and activities of biofilms on metals and alloys typically have a significant impact on biocorrosion. By altering the chemical environment, microbial infections can impact electrochemical events at a metal surface [84]. Two pathogenic strains (B. subtilis and E. coli) were tested for antibacterial activity in vitro in this investigation, and the inhibition zone was then evaluated as indicated in Table 8. According to the findings, both TMP and TBF exhibit strong antibacterial properties. When it came to B. subtilis and E. coli, TBF demonstrated inhibition zones of 23 mm and 20 mm, respectively. In contrast, TMP exhibited slightly smaller but still effective zones of 21 mm and 19 mm. By contrast, the common antibiotic azithromycin created inhibition zones of 23 mm (B. subtilis) and 15 mm (E. coli). The antibacterial properties of the as-prepared inhibitors prevent bacterial growth and biofilm formation on the metal surface. This is a critical factor, as biofilms formed by bacteria can create localized microenvironments that accelerate corrosion processes. By inhibiting bacterial adhesion and biofilm development, the compounds indirectly reduce microbiologically influenced corrosion (MIC), a known contributor to material degradation. Therefore, these results suggest that the tested inhibitors exhibit significant antibacterial activity alongside potent dissolution inhibition capabilities. They are promising options for reducing carbon steel corrosion caused by both chemical and microbiological factors because of their dual activity. Such multifunctional behavior has been reported in the literature [85–88], highlighting its potential for practical applications.

**Table 8:** Antibacterial activity expressed as Inhibition zones in mm of the tested samples against pathogenic bacteria

Microorganisms	TBF	TMP	DMSO	Azithromycin
Gram-positive bacteria				
B. subtilis	23	21	-ve	23
Gram-negative bacteria				
E. coli	20	19	-ve	15

### DFT

**Fig. 15:**
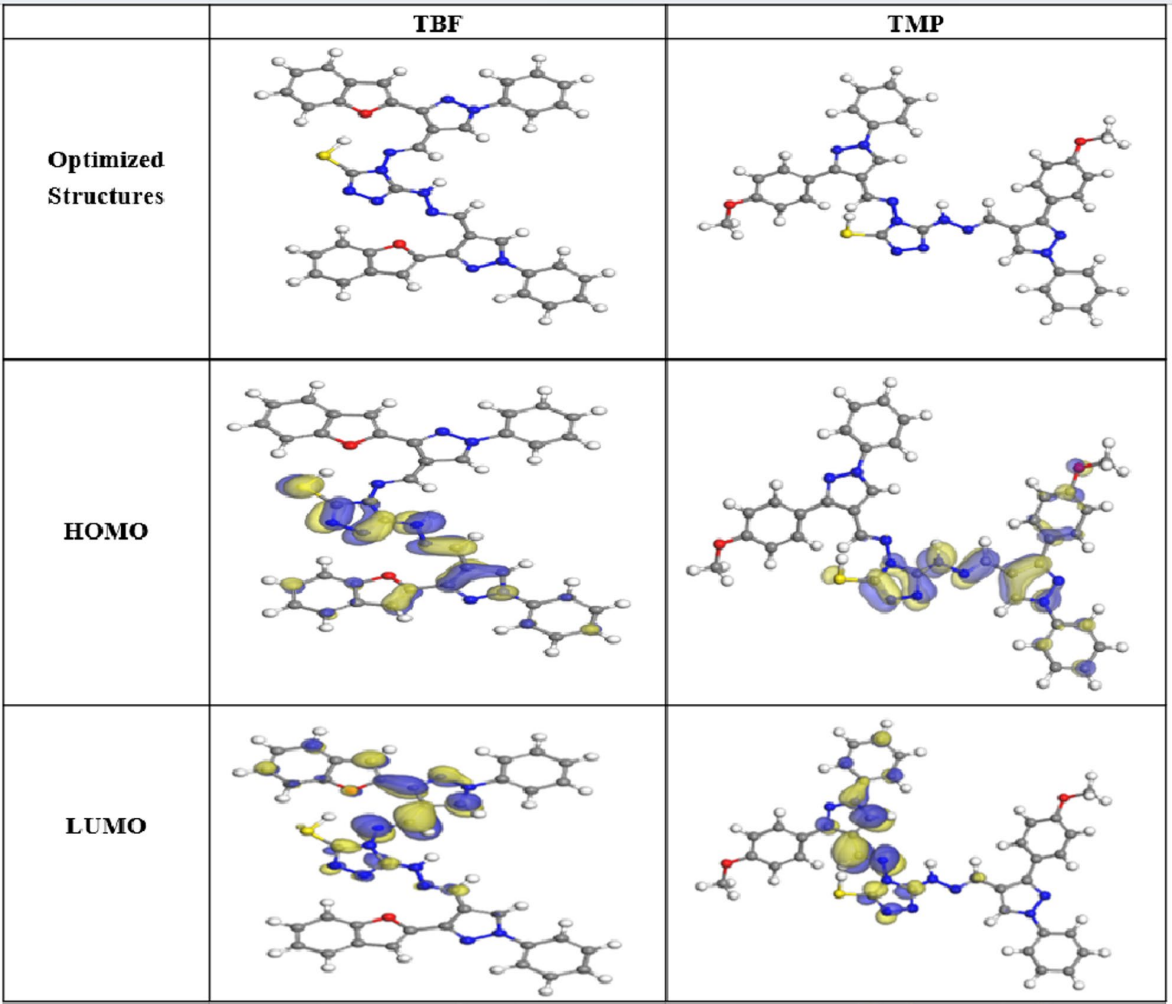
The optimized structure, HOMO, and LUMO for TBF and TMP

**Fig. 16:**
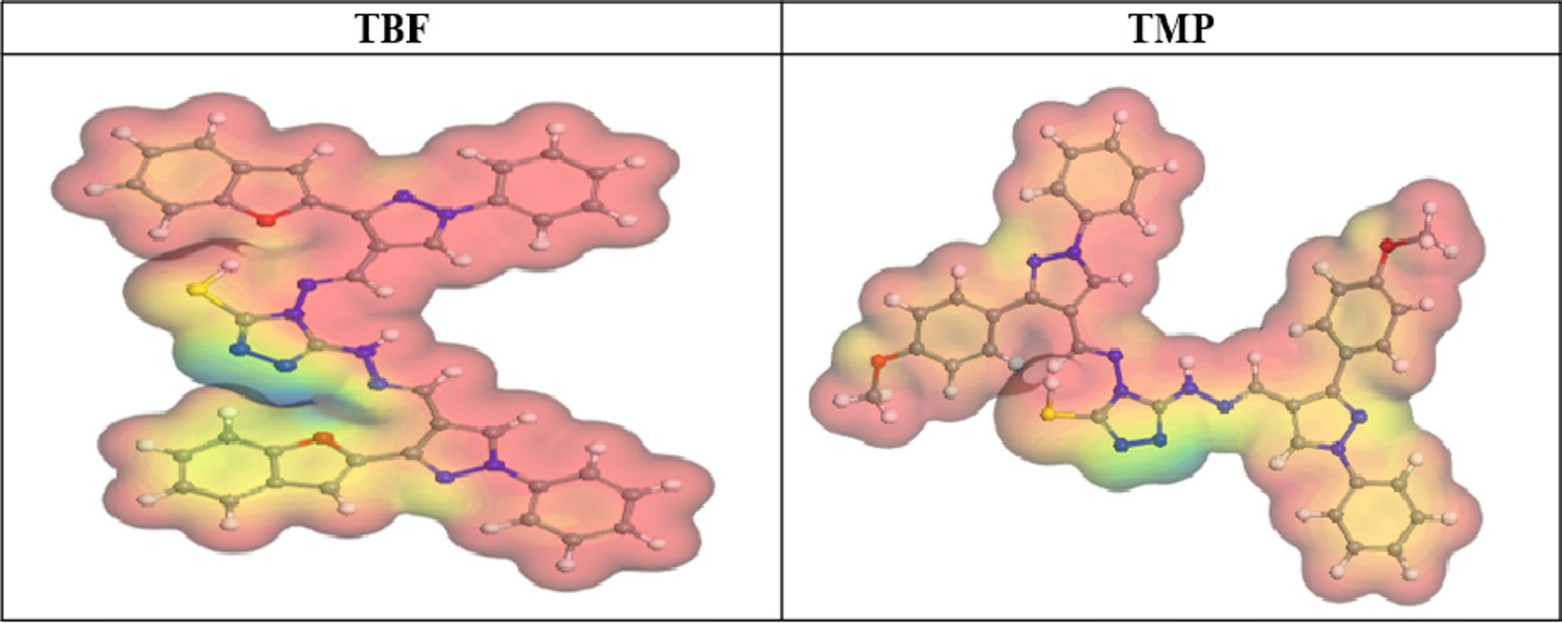
The MEP maps of TBF and TMP using the DMol^3^ module

The effectiveness of TBF and TMP as corrosion inhibitors for carbon steel can be assessed through theoretical investigations of their molecular structure and electronic properties. DFT offers valuable insights into the interaction between the inhibitor and the metal surface [89]. A summary of the optimized geometries and spatial distributions of the inhibitor molecules’ LUMO and HOMO is presented in Fig. 15; Table 9. Whereby the energies of HOMO and LUMO levels provide important information about a molecule’s capacity to give or receive electrons when interacting with a metallic surface, according to the Frontier Molecular Orbital (FMO) theory. These orbital energies largely determine the molecule’s possible anticorrosive properties [90]. The molecule’s ability to donate electrons is indicated by the quantum chemical characteristic known as HOMO [91]. An elevation in anticorrosive performance arises from an increase in HOMO value. A lower value of LUMO implies better receiving of electrons from the metal’s d orbital, creating a firm bond between the inhibitor and the metal through a donor-acceptor mechanism [92]. Based on Table 9, TBF exhibits the highest corrosion inhibition efficiency, which is attributed to its higher HOMO energy (-4.468 eV) and lower LUMO energy (– 2.334 eV) compared to the HOMO (-4.494 eV) and LUMO (– 2.264 eV) values of TMP [93]. An important metric for evaluating how well inhibitor substances work to impede corrosion processes is the energy difference (ΔE_gap_). A smaller energy gap (ΔE_gap_ = E_LUMO_ − E_HOMO_) is associated with a more effective inhibitor as it facilitates the electron transfer to the metallic surface. So, the inhibitor is more likely to provide electrons and successfully resist corrosion when the energy gap is less [94]. TBF compound has an ΔE of 2.13 eV, which is noticeably lower than the ΔE of 2.23 eV found for TMP, according to the data presented in Table 9. This demonstrates TBF’s greater propensity to adsorb onto the surface. Inhibitors can supply electrons to the carbon steel because they typically have moderately low electronegativity (χ) values. Stronger attachment to the interface is produced by inhibitors with higher χ, which are better able to take electrons from steel and return them [95]. According to Table 9, TBF and TMP molecules have high electronegativity values, which facilitate efficient electron acceptance and back-donation, strengthening their interaction with the steel. Hardness (ɳ) and softness (σ) must be considered while analyzing the stability, reactivity, and effectiveness of any selected inhibitor. When compared to molecules with high hardness and low softness, organic scaffolds with low hardness and high softness exhibit better anticorrosion qualities due to their tendency for effective electron transfer during adsorption [93]. Table 9 further shows that the TBF molecule exhibits better inhibitory efficacy and more efficient electron transport, with greater σ and lower η than TMP. Furthermore, TBF and TMP can deliver their electrons to the surface, as shown in Table 9, which shows that the acquired number of transferred electrons (ΔN) values for these inhibitors are less than 3.6 and greater than zero, according to the scientific literature [96]. Also, the literature states that ΔE_back−donation_ < 0 denotes an energetically favorable back-donation from the studied scaffolds toward the steel surface. Table 9 shows that ΔE_back−donation_ < 0, indicating that the charge delivered from TBF and TMP and subsequent back-donation from these molecules is energetically favorable [97]. In addition, dipole moment plays an important role in concluding how effective a compound is as a corrosion inhibitor. An increase in the dipole moment increases the energy needed for distortion and increases the adsorption of the investigated particles on the metal surface. Therefore, a higher inhibiting proficiency is correlated with a larger dipole moment [98]. According to Table 9, the TBF compound exhibits a greater dipole moment value (17.5485 debye) than TMP (8.7946 debye), suggesting a stronger propensity for adsorption on the surface. Furthermore, the propensity of inhibitor molecules to preserve the surface is correlated with their molecular surface area. The larger the molecular structure and the larger the interface area, the greater the inhibition efficacy. As shown in Table 9, TMP has the largest molecular surface area (676.61 Å²); however, this is inconsistent with experimental findings, as TBF has a greater inhibition proficiency than TMP. This may be because the TMP molecule has a high steric hindrance, which reduces its adsorption on the carbon steel [99]. In addition, TMP may adsorb in a less optimal tilted configuration that exposes more active sites, while TBF molecules are expectedto adopt a more advantageous flat-lying orientation during adsorption, optimizing surface coverage and improving film density. Molecular Electrostatic Potential (MEP) analysis is widely employed for locating organic molecules’ active sites. It entails analyzing how the electron density is distributed over the molecule’s surface [4]. The possible nucleophilic and electrophilic regions inside the molecules were identified *via* MEP maps. On the MEP map in Fig. 16, nucleophilic and electrophilic sites are indicated by red and blue patches, respectively [100]. Examination of Fig. 16 reveals that most of the negative contributions are located over the whole structure. Conversely, Positive potential is localized on the triazole nitrogen atoms [101]. It is possible that the inhibitor species’ red zones, which have a higher electron density, indicate the best locations for interactions with the surface to create a strong adsorbed protective film.

**Table 9:** Theoretical parameters calculated for TBF and TMP

Quantum parameters	TBF	TMP
E_HOMO,_ eV	– 4.468	– 4.494
E_LUMO,_ eV	– 2.334	– 2.264
ΔE = E_LUMO_-E_HOMO_, eV	2.13	2.23
I	4.47	4.49
A	2.33	2.26
Χ	3.40	3.38
ɳ	1.07	1.12
σ	0.94	0.90
ΔN	1.69	1.62
ΔE_back−donation_	– 0.27	– 0.28
dipole moment	17.5485	8.7946
Molecular surface area, Å^2^	661.152037	676.612418

### MC simulations

**Fig. 17:**
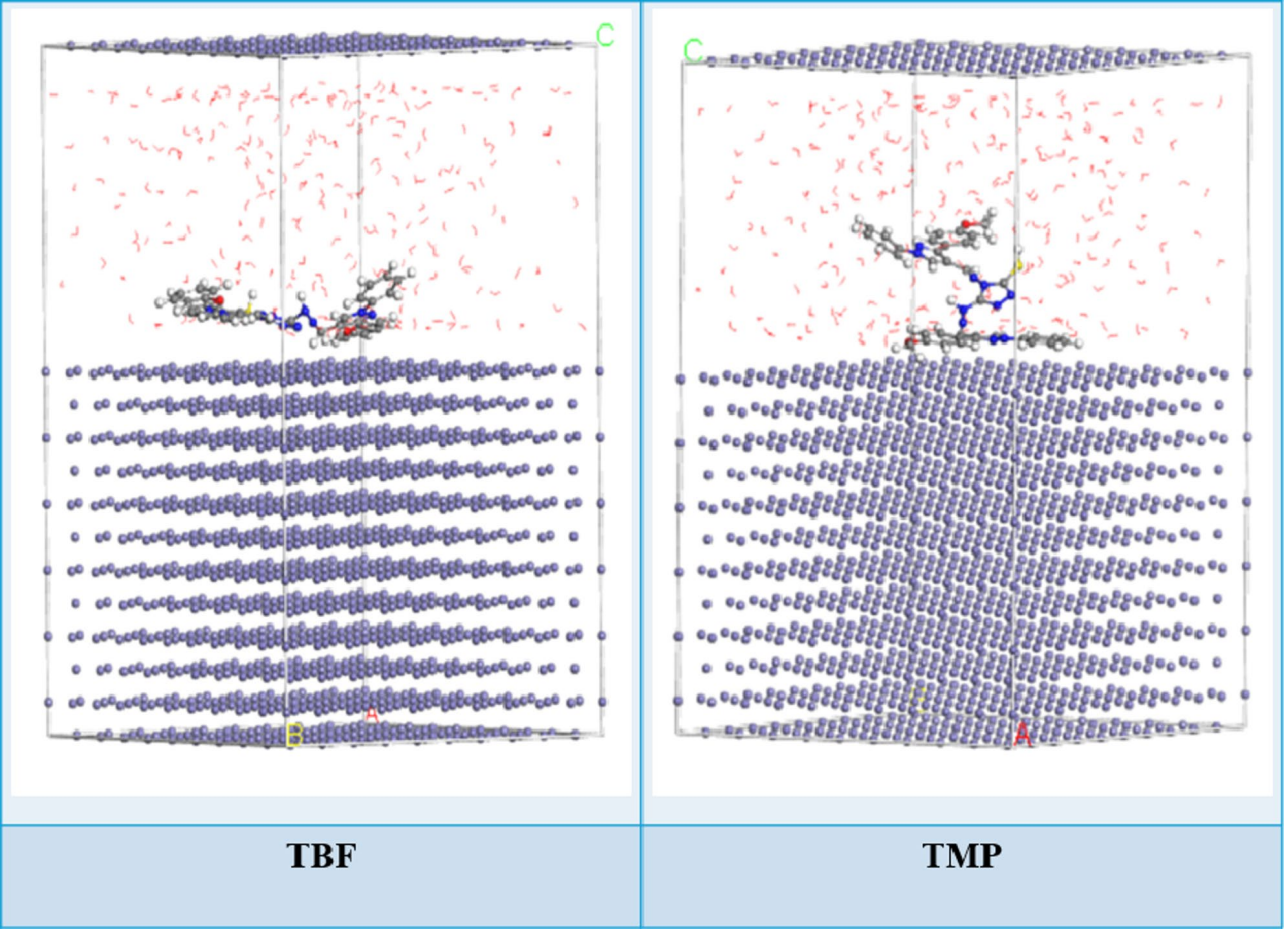
The most suitable configuration for the adsorption of TBF and TMP on the Fe (1 1 0) substrate

MC simulations of TBF and TMP molecules gave the configurations presented in Fig. 17. Both TBF and TMP molecules adsorb almost parallel to the Fe (110(, as seen in Fig. 17. In contrast to TMP, TBF has a flatter orientation and is situated nearer the metal surface. This configuration reduces the available surface area for corrosive assault by strengthening the contact and encouraging the creation of a more compact and protective adsorbed layer. Table 10 summarizes the computed MC data, which shows a negative sign for the adsorption energy, indicating a strong and spontaneous interaction between the inhibitors and Fe (110) [102]. TBF molecule (-3493.28 kcal mol^− 1^) exhibited a greater negative adsorption energy than the TMP molecule (-3390.53 kcal mol^− 1^), according to Table 10. This result suggests that the TBF molecule exhibits greater adsorption on Fe (110), suggesting the creation of a persistent adsorbed layer that successfully reduces carbon steel corrosion and supports experimental findings [103].

Additionally, Table 10 shows that the TBF molecule’s unrelaxed adsorption energy is higher than that of the TMP molecules. In a similar vein, the TBF molecule’s adsorption energy values in its relaxed form remained higher than those of the TMP following geometry optimization. These findings support the TBF molecule’s superior corrosion-resistant properties when compared to TMP. The amount of energy required to remove one of the adsorbed molecules is known as the conformational energy (dE_ads_/dN_i_) between the substrate and adsorbate [104]. TBF has a larger

dE_ads_/dN_i_ value than TMP, according to the findings shown in Table 10. This suggests that TBF is more capable of adsorption than TMP. Additionally, the dE_ads_/dN_i_ value for water is lower than that of the investigated inhibitors, suggesting that the inhibitors were adsorbed on the carbon steel surface more firmly than water molecules, supporting the substitution of inhibitors for water molecules [105]. Additionally, it can be summed up that these MC results agree well with both the experimental data and the DFT computations.

**Table 10:** Data calculated by the MC simulation for the adsorption of TBF and TMP on Fe(110)

Structures	Adsorption energy/ kcal mol^− 1^	Rigid adsorption energy/ kcal mol^− 1^	Deformation energy/ kcal mol^− 1^	dE_ads_/dN_i_: Inhibitor, kcal mol^− 1^	dE_ads_/dN_i_: Water, kcalmol^− 1^
Fe (1 1 0) TBF Water	– 3493.28	– 3689.33	196.04	– 303.08	– 12.44
Fe (1 1 0) TMP Water	– 3390.53	– 3554.03	163.50	– 226.14	– 13.24

### PZC and proposed adsorption mechanism

The sign of the surface charge on carbon steel immersed in H_2_SO_4_ can be assessed by comparing the values of E_PZC_ and E_OCP_ using the corresponding relation [106]:

E_r_ = E_OCP_ - E_PZC_ (11).

**Fig. 18:**
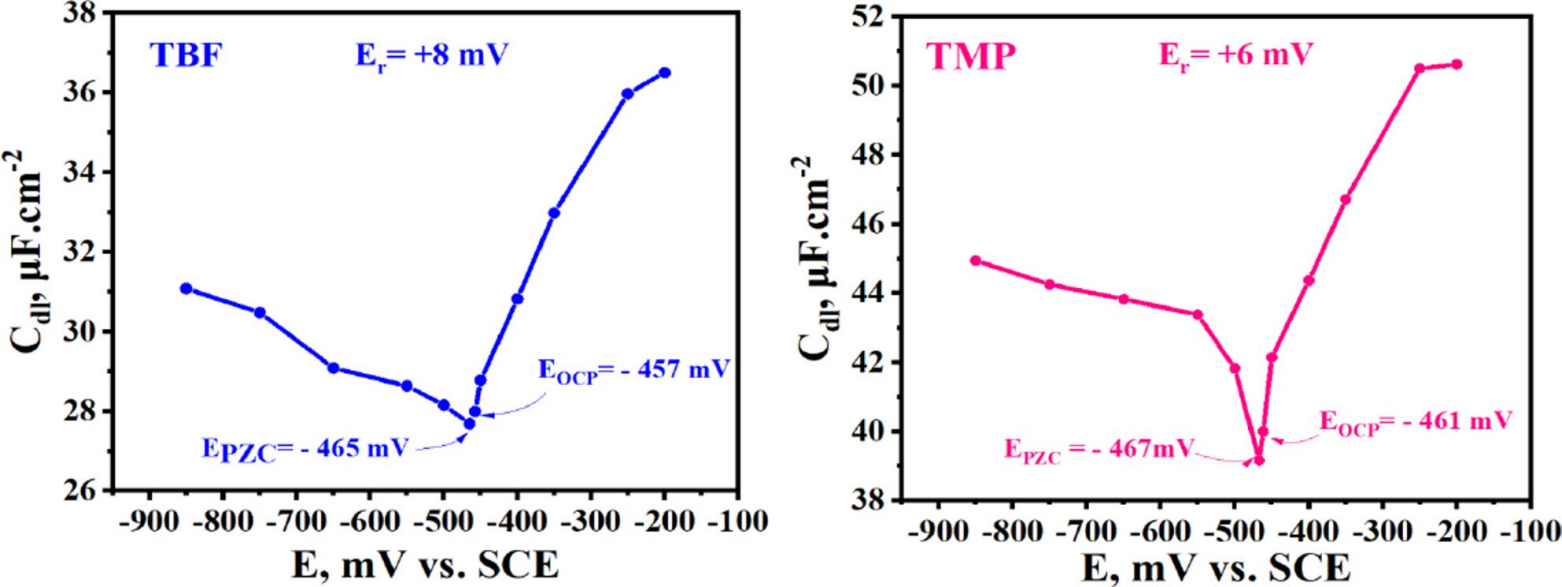
PZC of carbon steel measured in 0.5 M H_2_SO_4_ solution with 5 × 10^− 5^ M of TBF and TMP

The surface affinity of carbon steel for ionic ions in the corrosive media is reflected in the E_r_. A positive E_r_ shows a propensity for anion adsorption, whereas a negative E_r_ suggests a surface tendency to adsorb cations. The PZC of carbon steel at room temperature in a solution with 5 × 10^− 5^ M of TBF and TMP, respectively, in 0.5 M H_2_SO_4_, is displayed in Fig. 18. The point of zero charge (mV) reaches its minimum at -465 and − 467 mV for TBF and TMP, respectively, and the potential profile shows a parabolic pattern. The fact that the carbon steel surface has a positive charge in these circumstances is confirmed by the positive value of E_r_, which is derived from Eq. (11) [39].

**Fig. 19:**
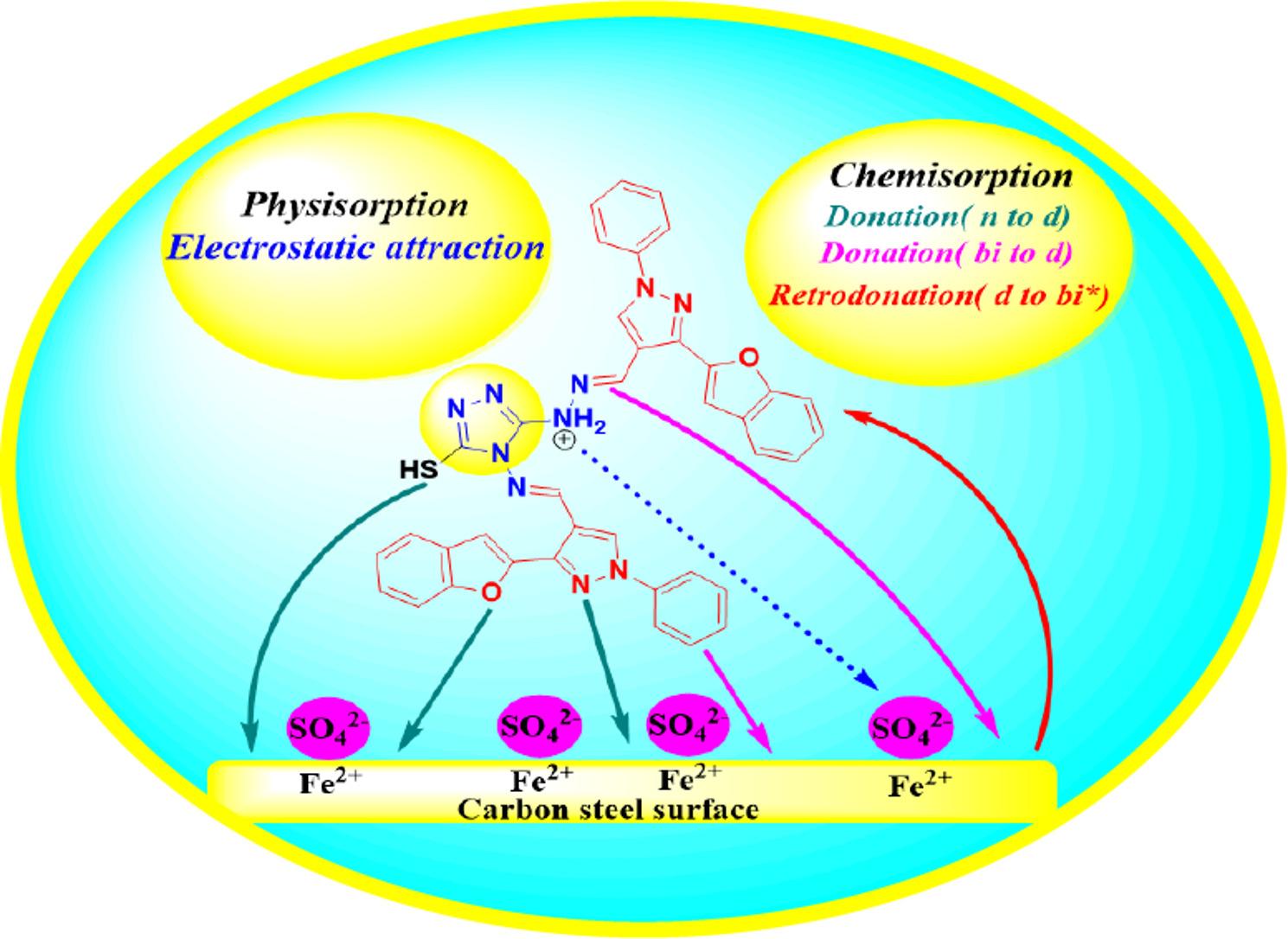
Possible adsorption mechanism of TBF

Based on electrochemical tests, DFT, and MC simulations, the proposed mechanism can be discussed. SO_4_^2−^ions in H_2_SO_4_ solutions may preferentially adsorb first, adding extra negative charges to the surface and encouraging more inhibitor cation adsorption. By physically adhering to the negative SO_4_^2−^ ions, the protonated TBF and TMP molecules can prevent further corrosive ions from migrating toward the inner surface. On the other hand, the increase in IE% with temperature indicates the inclination of TBF and TMP for chemical adsorption on the carbon steel surface [66]. This occurs when lone pairs or π-electrons are donated to the metal’s unoccupied d orbitals by the heteroatoms or delocalized orbitals of the inhibitor. Retro-donation, involving the migration of electrons to the antibonding molecular orbitals of TBF and TMP, represents another mechanism by which the inhibitors can cling to metal surface [107]. These interactions greatly increase carbon steel’s corrosion resistance in an aggressive acid solution by fostering the development of a compact protective coating that successfully thwarts corrosive attack [108]. Figure 19 shows a schematic representation of the TBF’ suggested dissolution inhibition mechanism concerning the carbon steel surface.

### Comparative studies with previous reports

The synthesized inhibitors, TBF and TMP, exhibited excellent corrosion inhibition performance for carbon steel in H_2_SO_4_, comparable to or even superior to other reported analogues in Table 11. The main advantages of our compounds arise from the strategic incorporation of multiple active moieties, including pyrazole, triazole, benzofuran (in TBF), and methoxy (in TMP) within a single molecular framework. This structural design provides a synergistic electronic effect that enhances adsorption onto the carbon steel surface and improves corrosion inhibition efficiency even at low concentrations. The remarkable inhibitory performance of TBF and TMP therefore confirms the effectiveness of this molecular design compared to previously reported pyrazole–triazole derivatives.

**Table 11:** Comparison of the corrosion Inhibition performance of pyrazole and Triazole derivatives reported in the literature with those developed in the present work

Inhibitors	Corrosive Medium	Conc. of inhibitor (M)	% IE		Sample	Reference
			PP	EIS		
3- (3,5-dimethyl-1 H-pyrazol-1-yl)-1 H-1,2,4-triazole (TzPz1)	1.0 M HCl	10^− 3^	92.6	92.1	Carbon steel	[72]
3-(3,5- dimethyl-1 H-pyrazol-1-yl)-1-methyl-1 H-1,2,4-triazole (TzPz2)			95.2	95.4		
ethyl 2-(3-(3,5-dimethyl-1 H-pyrazol1-yl)-H-1,2,4-triazol-1-yl) ethan-ol(TzPz)	1.0 M HCl	10^− 3^	91.9	90.2	Carbon steel	[109]
ethyl 2-(3-(3,5-dimethyl-1 H-pyrazol-1 yl)-1 H-1,2,4-triazol-1-yl)acetate(TzPzAcEt)	1.0 M HCl	10^− 3^	93.9	94.4	Carbon steel	[110]
2-(3-(3,5-dimethyl 1 H-pyrazol-1-yl)-1 H-1,2,4-triazol-1-yl)acetic acid (TzPzAcid)			91.6	91.3		
1-(3-phenyl-4,5-dihydropyrazol-1-yl)-2-(1 H-1,2,4-triazol-1-yl) ethanone(BHOT)	1.0 M HCl	10^− 3^	98	98	Mild steel	[111]
1-(3-(4-fluorophenyl)-4,5-dihydropyrazol-1-yl)-2-(1 H-1,2,4-triazol-1-yl) ethanone (FHOT)			98	97		
1-(3-(2,4-dichlorophenyl)-4,5-dihydropyrazol-1-yl)-2-(1 H-1,2,4-triazol-1-yl) ethanone (CHOT)			98	99		
(Z)-4-amino-5-(2-(pyridin4-ylmethylene)hydrazinyl)-4 H-1,2,4-triazole-3-thiol (KB1)	0.5 M H_2_SO_4_	9 × 10^− 5^	87.5	86.9	Carbon steel	[112]
(Z)-4-amino-5-(2-((2-azidoquinolin-3-yl) methylene)hydrazinyl)-4 H-1,2,4-triazole-3-thiol (KB2)			91.4	92.4		
Ethyl 1-amino-3-(2-chlorophenyl)-5,10-dioxo-5,10-dihydro-1 H-pyrazolo[1,2-b] phthalazine-2 carboxylate (Py-1)	2.0 M H_2_SO_4_	10^− 3^	96.8	96.7	stainless-steel	[113]
Ethyl 1-amino-5,10- dioxo-3-(p-tolyl)-5,10-dihydro-1 H-pyrazolo[1,2-b] phthalazine-2- carboxylate (Py-2)			91.2	90.9		
4,4’-(phenylmethylene)bis (3-methyl-1- phenyl-1 H-pyrazol-5-ol) [PYR-H]	1.0 M HCl	3.66 × 10^− 4^	86.26	84.12	Mild steel	[114]
4,4’-((4-nitrophenyl)methylene)bis (3-methyl-1-phenyl-1 H-pyrazol-5-ol) [PYR-NO_2_]			88.28	86.27		
4,4’-((4- hydroxyphenyl) methylene)bis (3-methyl-1-phenyl-1 H-pyrazol-5-ol) [PYR-OH]			91.16	86.86		
4,4’-((2-hydroxy-4-methox-yphenyl)methylene)bis (3- methyl-1-phenyl-1 H-pyrazol-5-ol) [PYR-OHOMe]			94.03	91.06		
(Z)-4-((4- methoxybenzylidene)amino)-5-methyl-2,4-dihydro-3 H-1,2,4-triazole-3-thione (2 C)	1.0 M HCl	10^− 3^	83.0	86.0	Carbon steel	[115]
4-(((*E*)-(3-(benzofuran-2-yl)-1-phenyl-1*H*-pyrazol-4-yl)methylene)amino)-5-(2-((*E*)-(3-(benzofuran-2-yl)-1-phenyl-1*H*-pyrazol-4-yl)methylene)hydrazinyl)-4*H*-1,2,4-triazole-3-thiol (TBF)	0.5 M H_2_SO_4_	9 × 10^− 5^	95.3	94.8	Carbon steel	Our work
4-(((*E*)-(3-(4-methoxyphenyl)-1-phenyl-1*H*-pyrazol-4-yl)methylene)amino)-5-(2-((*E*)-(3-(4-methoxyphenyl)-1-phenyl-1*H*-pyrazol-4-yl)methylene)hydrazinyl)-4*H*-1,2,4-triazole-3-thiol (TMP)			93.5	93.4		

## Conclusion

Two newly synthesized compounds, abbreviated as TBF and TMP, were characterized and tested as inhibitors for the dissolution of carbon steel in 0.5 M H_2_SO_4_ via a variety of measuring techniques involving electrochemical methods, DFT, MC, and surface morphology studies. At the higher dose, TBF and TMP exhibited inhibition efficiencies of 95.3% and 93.5% according to the pp technique, indicating a mixed type of inhibitory behavior. EIS verified that the carbon steel reaction mechanism was governed by the charge transfer process, and corrosion could be avoided by the adsorbed layer of TBF and TMP on the surface. The data acquired from EIS correlate very well with those obtained from PP. Based on EIS, higher concentrations of TBF and TMP result in a significant increase in R_ct_ while simultaneously decreasing the C_dl_, confirming enhanced corrosion inhibition. Strong adsorptive interactions among the inhibitors and carbon steel are indicated by studying the role of temperature on the adsorption of TBF and TMP over a range of 298–328 K. The inhibition efficiency increased with temperature, reaching a maximum at 318 K, followed by a slight decline at 328 K. This suggests that TBF and TMP undergo desorption at 328 K, reducing their protective effect. Following the Langmuir adsorption isotherm, TBF and TMP underwent spontaneous adsorption. TBF and TMP are optimally bound on the carbon steel, as indicated *via* instrumentalexaminations conducted using AFM, FT-IR,contact angle, and XPS analysis. The capacity of TBF and TMP to combine with Fe^2+^ ions to form complexes and lessen metal dissolution in acidic environments has been established. TBF and TMP exhibited notable antibacterial effects against B. subtilis and E. coli in addition to their efficacy as inhibitors for carbon steel in acidic situations. Their potential as multipurpose agents for use in environments where both microbial contamination and metal corrosion are issues is highlighted by this dual performance. The carbon steel surface is positively charged based on PZC. The DFT-derived parameters and MC simulations demonstrated that the TBF has greater corrosion-inhibitory efficacy than TMP and showed strong consistency with the experimental results. The superiority of the tested compounds is clearly demonstrated by the comparative data in Table 11, which shows that their inhibition efficiencies consistently exceed those reported for structurally related compounds under similar acidic conditions. This superior performance can be attributed to the novel synergistic molecular architecture of our compounds, which provides optimal surface coverage and binding strength. In addition, the ultrasound-assisted synthesis route provides a greener and more sustainable alternative to traditional reflux methods. Overall, the synthesized compounds exhibit excellent potential as efficient and eco-friendly corrosion inhibitors, particularly suitable for industrial applications where carbon steel is exposed to highly acidic environments.

## Supplementary Information

Below is the link to the electronic supplementary material.

Supplementary Material 1.

## Data Availability

All data and analysis during this study are available in this article and its supplementary file.
