# Supplementary material for: Novel pyrazole-clubbed triazole scaffolds as promising inhibitors for carbon steel corrosion in sulfuric acid and as antibacterial agents: electrochemical and computational evaluation
Source: BMC Chem. 2026 Jan 31;20(1):37. doi: 10.1186/s13065-025-01704-x (PMC12931072; doi:10.1186/s13065-025-01704-x)
Supplement: Supplementary file 1 — Supplementary Material 1. [file 13065_2025_1704_MOESM1_ESM.docx]

Novel Pyrazole-Clubbed Triazole Scaffolds as Promising Inhibitors for Carbon Steel Corrosion in Sulfuric Acid and as Antibacterial Agents: Electrochemical and Computational Evaluation.

Kamelia Belal^1,2^, A.H. El-Askalany^1^, Eslam A Ghaith^1,3^, Ahmed Fathi Salem Molouk^*1,2,3^

^1^Department of Chemistry, Faculty of Science, Mansoura University, Mansoura 35516, Egypt

^2^ Mansoura University Sustainable Energy Research Lab (MSER), Faculty of Science, Mansoura University, Mansoura 35516, Egypt

^3^Faculty of Science, New Mansoura University, New Mansoura City, Egypt

^*^ Correspondence author: Ahmed Fathi Salem Molouk

E-mail address: [molouk82@mans.edu.eg](mailto:molouk82@mans.edu.eg)

Supplementary Material

**General experimental:**

Melting points (uncorrected) were measured using a Gallenkamp melting-point apparatus and were uncorrected. The reaction mixture was monitored using thin-layer chromatography (TLC), which was performed on silica gel 60 F_254_ precoated aluminum sheets and visualized under ultraviolet (UV) light. Also, Infrared (IR) spectra were recorded on a JEOL FT-IR Spectrometer (Reflectance). A Bruker 400 M*Hz* or JEOL 500 M*Hz* spectrometers were used to record ^1^H-NMR and ^13^C-NMR spectra in the presence of DMSO-*d_6_* as solvent. Electron impact mass spectra were determined at 70 eV on a Varian MAT 3311 Kratos instrument (Micro-analytical center, Faculty of Science, Cairo University. Sonication was performed in a “Spectra lab model UCB 40D Ultrasonic cleaning bath” with a frequency of 40 kHz and power of 250 W. All chemicals and solvents were used as received from Sigma Aldrich and Fisher Scientific companies

Figure caption

**S1**. ^1^H-NMR spectrum of compound TBF.

**S2**. ^13^C-NMR spectrum of compound TBF.

**S3**. Mass spectrum of Compound TBF.

**S4**.^1^H-NMR spectrum of compound TMP.

**S5**. ^13^C-NMR spectrum of compound TMP.

**S6**. Mass spectrum of compound TMP.

**S1**. ^1^H-NMR spectrum of compound TBF.

**S2**. ^13^C-NMR spectrum of compound TBF.

**S3**. Mass spectrum of Compound TBF.

**S4**.^1^H-NMR spectrum of compound TMP.

**S5**. ^13^C-NMR spectrum of compound TMP.

**S6**. Mass spectrum of compound TMP.
